# Proteomics Recapitulates Ovarian Proteins Relevant to Puberty and Fertility in Brahman Heifers (*Bos indicus* L.)

**DOI:** 10.3390/genes10110923

**Published:** 2019-11-12

**Authors:** Muhammad S. Tahir, Loan T. Nguyen, Benjamin L. Schulz, Gry A. Boe-Hansen, Milton G. Thomas, Stephen S. Moore, Li Yieng Lau, Marina R. S. Fortes

**Affiliations:** 1 School of Chemistry and Molecular Bioscience, University of Queensland, Brisbane 4072, Queensland, Australia; m.tahir@uqconnect.edu.au (M.S.T.); b.schulz@uq.edu.au (B.L.S.); liyieng.lau@uq.net.au (L.Y.L.); 2 Queensland Alliance for Agriculture and Food Innovation, University of Queensland, Brisbane 4072, Queensland, Australia; t.nguyen3@uq.edu.au (L.T.N.); s.moore3@uq.edu.au (S.S.M.); 3 School of Veterinary Sciences, University of Queensland, Brisbane 4343, Queensland, Australia; g.boehansen@uq.edu.au; 4 Department of Animal Science, Colorado State University, Fort Collins, CO 80523, USA; milt.thomas@colostate.edu

**Keywords:** *Bos indicus*, ovary, puberty, steroidogenesis, progesterone signaling, oocyte maturation, *corpus luteum*, massspectrometry

## Abstract

High fertility and early puberty in *Bos indicus* heifers are desirable and genetically correlated traits in beef production. The hypothalamus–pituitary–ovarian (HPO) axis synthesizes steroid hormones, which contribute to the shift from the pre-pubertal state into the post-pubertal state and influence subsequent fertility. Understanding variations in abundance of proteins that govern steroid synthesis and ovarian signaling pathways remains crucial to understanding puberty and fertility. We used whole ovaries of six pre-pubertal and six post-pubertal Brahman heifers to conduct differential abundance analyses of protein profiles between the two physiological states. Extracted proteins were digested into peptides followed by identification and quantification with massspectrometry (MS) by sequential window acquisition of all instances of theoretical fragment ion mass spectrometry (SWATH-MS). MS and statistical analysis identified 566 significantly differentially abundant (DA) proteins (adjusted *p* < 0.05), which were then analyzed for gene ontology and pathway enrichment. Our data indicated an up-regulation of steroidogenic proteins contributing to progesterone synthesis at luteal phase post-puberty. Proteins related to progesterone signaling, TGF-β, retinoic acid, extracellular matrix, cytoskeleton, and pleiotrophin signaling were DA in this study. The DA proteins probably relate to the formation and function of the *corpus luteum*, which is only present after ovulation, post-puberty. Some DA proteins might also be related to granulosa cells signaling, which regulates oocyte maturation or arrest in ovaries prior to ovulation. Ten DA proteins were coded by genes previously associated with reproductive traits according to the animal quantitative trait loci (QTL) database. In conclusion, the DA proteins and their pathways were related to ovarian activity in *Bos indicus* cattle. The genes that code for these proteins may explain some known QTLs and could be targeted in future genetic studies.

## 1. Introduction

Early puberty, pregnancy maintenance, and successful calving contribute to the life-time reproductive performance of cows [1]. *Bos indicus* cattle are tropically adapted but come across fertility problems in early reproductive age which include delayed puberty as compared to *Bos taurus* and pregnancy-related losses [2]. Age at puberty, as measured by observance of the first *corpus luteum* (CL) and age at first calving (AFC) are early in life reproductive traits that are crucial to heifers’ fertility [3,4,5,6,7,8]. Previous genome wide association studies have indicated the polygenic nature of these traits [9,10]. Fertility-related traits are expressed as a consequence of puberty, so it is important to investigate the biology of puberty as it impacts on subsequent fertility. 

Puberty begins with widespread changes in the hypothalamus–pituitary–ovarian (HPO) axis and plasma hormonal profiles [11,12]. Ovaries play a crucial role in the regulation of the HPO axis by negative and positive feedback mechanisms related to the gonadostat theory and the onset of puberty [13]. According to the gonadostat theory, ovarian estrogen imparts its negative feedback on the hypothalamus to suppress frequency of gonadotrophic releasing hormone (GnRH) pulses, which suppresses luteinizing hormone (LH) secretion from the pituitary in pre-pubertal life [14]. At puberty, estrogen through its positive feedback increases the pulse frequency of GnRH from hypothalamus, which releases LH and follicle stimulating hormone (FSH) from the pituitary, leading to pre-ovulatory follicle development and ovulation [11,13]. Ovarian estrogen feedback mechanisms are important in the complex systemic changes that control puberty.

Steroid synthesis in ovaries is dependent on the interconnected functioning of theca and granulosa cells in response to FSH and LH hormones. In bovine ovaries, theca cells synthesize androgen while follicular cells synthesize estrogen [15]. LH is the hormone signal for ovulation and therefore controls the luteinization of theca and granulosa cells that results in synthesis of progesterone by up-regulation of P450 enzymes *CYP11A* and *HSD3β1* and down-regulation of P450 enzyme *CYP17A1* and aromatase (*CYP19A1*) [16]. Progesterone is involved in oocyte quality and embryo development in cows [17] and its post-ovulatory rise, produced by the CL, is necessary for normal maintenance of pregnancy, meaning it contributes to heifer fertility [1].

The onset of puberty corresponds to the maturation of oocytes, which are arrested in the diplotene stage of meiosis prophase-1 in fetal life [18]. Ovarian granulosa and theca cells are crucial to oocyte maturation [19]. Oocyte maturation impacts on subsequent fertility, although its mechanisms are not entirely clear in cattle. Multiple studies have suggestedthe role of gonadotropins (LH/FSH) in inducing bovine oocyte meiotic maturation with probable dependence on paracrine functioning of growth factors (TGF-β, TGF-α, EGF, IGF-1, and activin) from theca and granulosa cells [20,21,22]. Progesterone also plays role in oocyte maturation [23,24]. Progesterone signals to rapid induction of oocyte maturation by increased intracellular Ca^2+^, inactivating the adenylate cyclase PKA system, initiating the Mos/MEK/MAPK cascade, and activating Cdk1/Cyclin-B, as reported in Xenopus frogs [25]. Granulosa cells are important for oocyte maturation, fertilization, and embryo development [26]. The interplay between granulosa cells and progesterone signaling occurs at two different stages of the estrous cycle to execute two important functions: (1) oocyte maturation just before ovulation and (2) CL activity after conversion of granulosa cells to luteal cells that will support subsequent pregnancy.

The physiological functions of tissues depend on the regulation of protein abundance, affecting biological pathways. The multifaceted phenomenon of puberty, including the production of progesterone in ovaries post-puberty, requires further elucidation that can be achieved with proteomics. Determination of protein differential abundance in ovarian tissues of pre-pubertal and first cycle post-pubertal heifers can be useful to revealing key players controlling the complex mechanisms of CL formation and puberty. Most studies done so far on ovarian functional pathways have addressed a limited number of candidate genes or proteins [27,28]. Global gene expression (transcriptomics) in ovaries of pre- and post-pubertal heifers have been reported, with many genes being differentially expressed between these two physiological states [28,29,30]. Transcriptome and proteome analyses often reveal different aspects of a physiological event [31]. Proteome analyses measure the effectors of biological function, i.e., the proteins themselves. A recent study identified neuropeptides that differed between pre- and post-pubertal cows in the hypothalamus and the pituitary [32]. A similar report for ovarian tissue is lacking. The aim of this study was to identify proteins and pathways that are related to the onset of puberty and are important for CL function, and which therefore could play a role in subsequent fertility. To achieve this goal, protein abundance was measured in the ovaries of pre-pubertal and post-pubertal heifers (at the luteal phase). 

## 2. Materials and Methods

### 2.1. Animal Selection and Sampling

All experimental procedures were approved by the Animal Ethics Committee of The University of Queensland, Production and Companion Animal Group (certificate number QAAFI/279/12). Twenty Brahman heifers were sourced as weanlings (<250 kg) from two commercial herds in Queensland, Australia, and managed together at the Gatton Campus facilities of the University of Queensland.

Heifers’ ovarian activity was observed using ultrasonography every fortnight for eight consecutive months (HS-2000(VET), Honda Electronics Inc.). When a CL was identified the heifer was deemed post-pubertal and paired randomly with a pre-pubertal heifer (no CL observed) for same-day euthanasia [3]. Euthanasia was planned for approximately 21 days after detection of the first CL, as described before [23]. On euthanasia day, we also measured the size of the largest follicle in each ovary and found that there was no statistical difference between pre- and post-pubertal heifers according to this criterium. The entire ovaries were harvested and preserved by snap freezing in liquid nitrogen and kept at −80 °C until protein extraction. We recorded which ovary had the CL for post-pubertal heifers. When thawed, the entire ovaries were pulverized and homogenized using a mortar and pestle and liquid nitrogen to produce a uniform sample for each ovary. Subsequently, aliquots of pulverized tissue of the left and right ovaries of the same heifer were mixed to produce a sample that would represent all ovarian tissue for proteomics. Plasma levels of progesterone were measured to confirm the luteal phase status post-puberty (average 2.0 ± 0.7 ng/mL). The average concentration of progesterone in pre-pubertal heifers was significantly lower (0.4 ± 0.2 ng/mL), as has been previously described [29].

### 2.2. Proteomics, Mass Spectrometry, and Data Analysis

Tissue samples from both ovaries were ground in liquid nitrogen so that fragments would represent the entire tissue. Subsequently, the buffer having 6M Guanidine chloride, 50 mM Tris pH 8, and 10 Mm DTT was added to lobind tubes containing samples. Samples were then sonicated at 35% for 10 s, and vortexed at 30 °C for 1 h in order to denature the proteins and break disulfide bonds. Then, 25 mM acrylamide was added to the samples and incubated at 30 °C for 1 h followed by the addition of 5 mM DTT. A small aliquot of the samples was precipitated by incubation at −20 °C for 16 h after adding four volumes of 1:1 methanol:acetone to one volume of sample. Afterwards, the proteins were precipitated from aliquot samples by centrifugation at 18,000 rcf for 10 min and the supernatant was discarded. The dried protein pellet was resuspended in 0.1% SDS. Protein concentration was measured using Nanodrop. Aliquots were further processed by filter-assisted sample preparation, adding 100 µg total protein to 10 kDa Cut-off Amicon columns which were then centrifuged at 18,000 rcf for 30 min. Ammonium bicarbonate buffer (50 mM) was added to the Amicon columns and centrifuged again using the same conditions as above. Ammonium bicarbonate buffer (50 mM) and trypsin were then added to the Amicon columns and incubated in a chamber of saturated humidity to prevent evaporation at 37 °C overnight. The Amicon columns were transferred to new tubes and centrifuged at 18,000 rcf for 30 min, followed by another centrifugation (same conditions) after addition of 0.5 M NaCl. The Amicon columns were discarded and the soluble peptides in the tubes were desalted using C18 ZipTips (Millipore) and resuspended in 9.25% acetonitrile and 0.1% formic acid. Desalted peptides were analyzed by liquid chromatography electrospray ionization tandem mass spectrometry (LC-ESI-MS/MS) with a TripleTof 5600 instrument as previously described [33], except in this case a 45 min LC gradient was used to separate the peptides. Proteins were identified using information-dependent acquisition analysis of one randomly chosen pre-puberty sample and one randomly chosen post-puberty sample with Protein Pilot v5.0.1 (SCIEX) blasted against a database of proteins downloaded from Uniprot on 28 March 2016, with a total of 43,813 entries assigned to *Bos taurus*, including 6870 reviewed entries from Swiss-prot and 36,948 unreviewed entries from TrEMBL. Sequential window acquisition of all theoretical mass spectra (SWATH)-MS relative quantitative proteomics [34] data was analyzed with PeakView v2.1 (SCIEX). Statistical analyses were performed using MSstats in R as previously described [35,36] to identify differentially abundant (DA) proteins with a *p* value lower than 0.05 adjusted for multiple testing [37].

### 2.3. Functional Enrichment and Protein Interaction Analysis

Enrichment analyses for gene ontology (GO) terms and biological pathways were performed using Uniprot accession identifiers of DA proteins as target lists and the identifiers of all detected proteins as our experimental background list using Database for Annotation, Visualization, and Integrated Discovery (DAVID) [38]. Protein-to-protein interaction analysis of specific proteins was done using Search Tool for the Retrieval of Interacting Genes/Proteins (STRING) [39]. 

### 2.4. Matching DA Proteins to Female Reproduction Quantitative Trait Loci (QTL)

The genes of DA proteins were also searched for their association with reproductive phenotypes according to the cattle quantitative trait loci database (Cattle QTLdb) [40]. Ovarian function is relevant to female reproduction in general and DA proteins from this study could be linked to CL function as well as puberty. Therefore, we performed an inclusive QTL analysis: we looked for all the female reproduction traits in the QTL database.

## 3. Results

**Figure 1:**
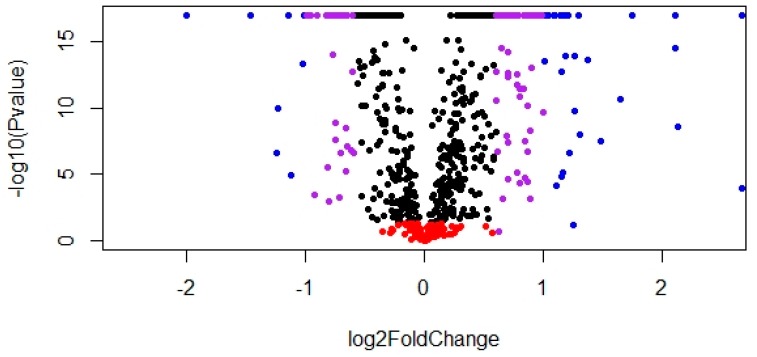
Volcano plot of differently abundant (DA) proteins in post-pubertal heifers at the luteal phase versus pre-pubertal Brahman heifers. Red: non-significant (adjusted *p* value > 0.05). Blue: log2 fold change > 1. Purple: log2 fold change > 0.6 and < 1. Black: log2 fold change > 0 and < 0.6.

Seven hundred and sixty-nine proteins were identified by LC-ESI-MS/MS detection and ProteinPilot database searching in the ovarian samples of both pre- and post-pubertal animals. Among the identified proteins, 566 were significantly DA when post- and pre-pubertal heifers were compared (Table S1). Among the DA proteins, 321 were up-regulated while 245 were down-regulated in post-pubertal heifers, as compared to pre-pubertal heifers (Figure 1). A high proportion (73%) of identified proteins were DA.

In our enrichment analyses using DAVID, no pathways or GO terms were significantly overrepresented in the DA gene list. However, when we analyzed the up- and down-regulated proteins as separated target lists, eight biological pathways terms were enriched: three in the up-regulated list of DA proteins, affecting different metabolic pathways, and five in the down-regulated list of DA proteins, affecting extracellular signaling through extracellular matrix and focal adhesion pathways (Table 1). The genes in each pathway are reported in the Supplementary Material (Table S2).

The 566 DA proteins were classified into 26 functional clusters using DAVID (Table 2 and Table S2). Functional clustering of DA proteins allowed the observation that most of proteins relevant to glycolysis, Tricarboxilic acid (TCA) cycle, pentose phosphate pathway, oxidative phosphorylation, cell redox homeostasis, and glutathione metabolism were up-regulated in post-pubertal heifers compared to pre-pubertal heifers. On the other hand, proteins directly or indirectly related to the extracellular matrix and focal adhesions were down-regulated in post-pubertal heifers (Table 2).

**Figure 2:**
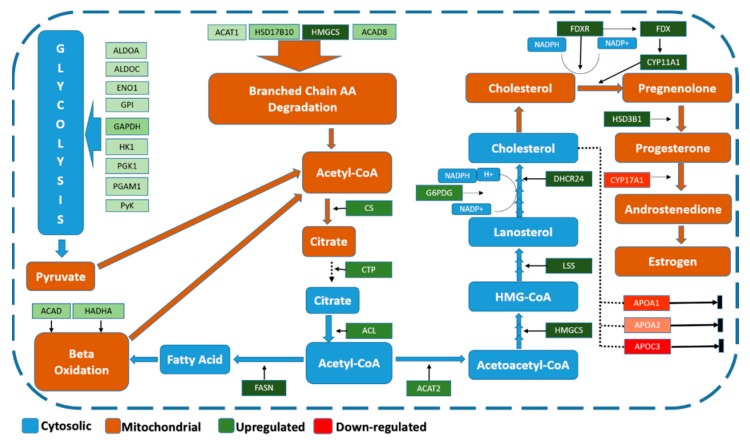
Proteins related to steroidogenesis differentially expressed in ovaries of post-pubertal heifers at the luteal phase compared to pre-pubertal Brahman heifers. Green and red tags indicate up- and down-regulated proteins, respectively, and their color intensity is related to the fold change level of proteins. Blue tags indicate literature-reported cytosolic compounds and processes. Brown tags indicate literature-reported mitochondrial compounds and processes. Fructose biphosphate aldolase-A (ALDOA). Fructose biphosphate aldolase-C (ALDOC). Alpha enolase (ENO1). Glycosylphosphatidyle inositol (GPI). Glyceraldehde-3-phosphate dehydrogenase (GAPDH). Hexokinase-1(HK1). Phosphoglycerate Kinase (PGK1). Phosphoglycerate mutase-1 (PGAM1). Pyruvate kinase (PyK). Acyl CoA dehydrogenase (ACAD). Hydroxyacyl-CoA dehydrogenase/3-ketoacyl-CoA thiolase/enoyl-CoA hydratase, alpha subunit (HADHA). Fattyacid synthase (FASN). ATP citrate lyase (ACL). Carboxylic acid transport protein (CTP). Citrate synthase (CS). Acetyle CoA acetyle transferase (ACAT2). Glucose-6-phosphate dehydrogenase (G6PGD). Acetyle CoA acetyle transferase-1 (ACAT1). 3-Hydroactle-CoA dehdrogenase-2 (HSD17B10). Hydromethylglutaryl-CoA synthase (HMGCS). Isobutaryl-CoA dehydrogenase (ACAD8). Lanosterole synthase (LSS). 24-Dehydrocholeaserole reductase (DHCR24). Ferridoxin reductase (FDXR). Ferridoxin (FDX). Cytochrome P450 family 11 subfamily A member 1 (CYP11A1). 3-beta-hdroxysteroid dehydrogenase (HSD3B1). Cytochrome P450 family 17 subfamily A member 1 (CYP17A1). Apolipoprotein-A1 (APOA1). Apolipoprotein-A2 (APOA2). Apolipoprotein-C3 (APOC3).

We observed the functional annotation of DA proteins, their associated GO terms, and pathways to manually curate protein interactions. Manual annotation of DA proteins allowed grouping into known interaction pathways that provide an overview of the ovarian proteome in the context of puberty. We identified a group of DA proteins associated with steroidogenesis (progesterone synthesis) at the luteal phase in post-pubertal heifers (Figure 2). The DA proteins related to progesterone synthesis were up-regulated in post-pubertal heifers compared to pre-pubertal heifers, which was exactly as expected since samples were collected at the luteal phase after puberty and a functioning CL was present. In this sense, up-regulated proteins post-puberty would be mostly from increased expression by CL cells. Meanwhile, the down-regulated proteins would correspond to proteins that had their expression reduced when granulosa cells were luteinized. 

**Figure 3:**
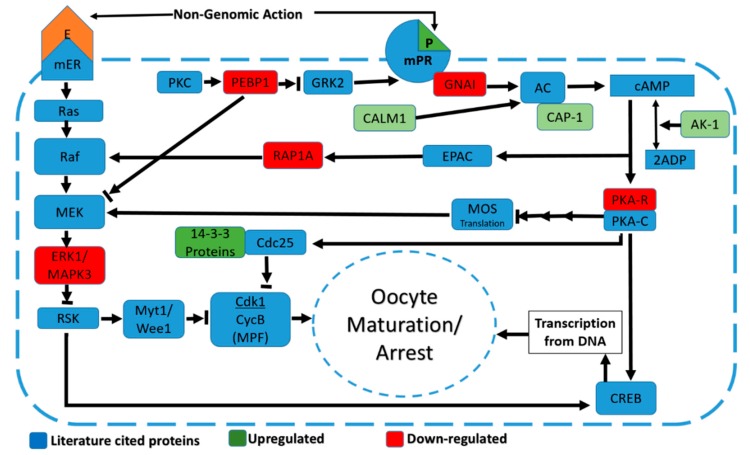
Differentially abundant proteins related to oocyte maturation/arrest through progesterone and MAPK signaling in ovaries of post-pubertal heifers at the luteal phase compared to pre-pubertal heifers. Green and red tags indicate up- and down-regulated proteins, respectively, and their color intensity is directly related to the fold change level of proteins. Blue tags indicate literature-reported proteins. Progesterone (P); Estrogen (E); Membrane progesterone receptor (mPR); Adenyl cyclase (AC); Cyclic adenosine monophosphate (cAMP); G-inhibitory protein (GNAI); Calmodulin-1 (CALM1); Adenlyl cyclase associated protein (CAP-1); Adenylate Kinase-1 (AK-1); Protein kinase-A regulatory subunit (PKAR); Protein kinase-A catalytic subunit (PKAC); Exchange factor directly activated by cAMP (EPAC); Ras related protein-1A (RAP1A); Mos protein (Mos); Cel division cycle protein 25 (Cdc25); Protein Kinase-C (PKC); Phosphatidylethanolanim binding protein (PEBP1); Beta adrenergic receptor kinase2 (GRK2); Membrane Estrogn receptor (mER); Mitogen activated protein kinase (MEK); Mitogen activated protein kinase-3 (MAPK3); Ribosomal S6 Kinase (RSK).; Myelin transcription factor-1 (Myt1); Wee1 like protein kinase (Wee1); Cell division kinase-1 (Cdk1).; Cyclin-B (CycB); cAMP responsive element binding protein (CREB).

Another group of DA proteins was related to regulation of oocyte maturation/arrest. Clustering by DAVID included nine proteins in progesterone-mediated oocyte maturation, estrogen, and cyclic adenine monophosphate (cAMP) signaling (Figure 3). The DA proteins from these clusters, when manually curated with evidence from the previous literature, seem involved in the regulation of oocyte maturation/arrest upon puberty in post-pubertal heifers. The DA proteins linked to oocyte maturation would be present in granulosa cells, which regulate and signal to promote oocyte maturation or arrest depending on the phase of the estrus cycle. Granulosa cells of the ovulatory follicle are luteinized and become CL cells post-puberty and at the luteal phase oocyte arrest is observed in the non-ovulatory follicles.

As an overall trend, the up-regulated proteins were involved in metabolic pathways and steroidogenesis while down-regulated proteins seemed to be involved in the regulation of CL function and oocyte maturation/arrest through the extracellular matrix and focal adhesion proteins in post-pubertal heifers at the luteal phase compared to pre-pubertal heifers (Table 3). Also, twenty-two DA proteins were identified as “uncharacterized” according to Uniprot (Table S1).

The processes involved in regulation of ribosome and translation were up-regulated, except for some specific down-regulated proteins like eukaryotic translation initiation factor-4B (EIF4B). The DA proteins involved in repression of transcription, such as various histones, LIM, and cysteine-rich domains protein-1 (LMCD1), hepatoma-derived growth factor (HDGF), and heterochromatin protein 1 binding protein-3 (HP1BP3), were down-regulated. Nine SERPINs, extracellular matrix proteins, were identified as DA, out of which eight were down-regulated in post-pubertal heifers compared to pre-pubertal heifers. Three of these proteins, SERPINE2, SERPINA3, and SERPINA3-3, were among the top twenty down-regulated proteins in our dataset (Table 3). This down-regulation of extracellular matrix (ECM) proteins may also be linked to the formation and function of the CL cells (see the discussion).

**Figure 4:**
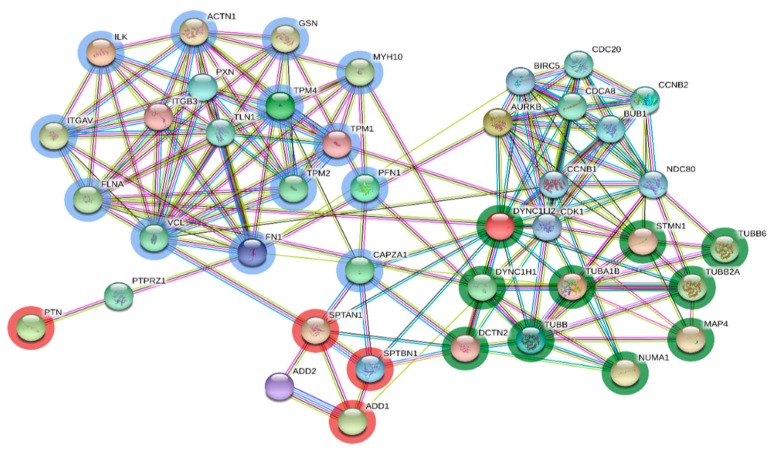
Protein-protein interaction for the subset of differentially abundant proteins involved in pleiotrophin signaling, focal adhesion, and cytoskeleton and microtubule processing under the influence of progesterone signaling. Blue circled nodes indicate proteins which are part of cytoskeleton and focal adhesion. Green circled nodes indicate proteins included in microtubule processing. Red circled nodes indicate proteins interacting with both blue and green nodes. Nomenclature of proteins is given in Table 1.

Analysis by STRING revealed interactions between proteins related to pleiotrophin signaling, cytoskeleton arrangement, focal adhesion, and spindle fiber arrangement (Figure 4). It is important to mention that pleiotrophin was a highly up-regulated protein in post-pubertal heifers at the luteal phase compared to pre-pubertal heifers. Pleiotrophin seems to play an important role while interacting with focal adhesion, cytoskeleton, and spindle assembly proteins during the onset of puberty and the establishment of progesterone signaling.

Using Uniprot ID information we were able to annotate the coding genes for all the DA proteins. We investigated the location of these genes to discover if they could be linked to any reproductive QTL. Ten DA proteins were coded by genes associated with reproductive traits according to QTL position (Table 4).

**Figure 5:**
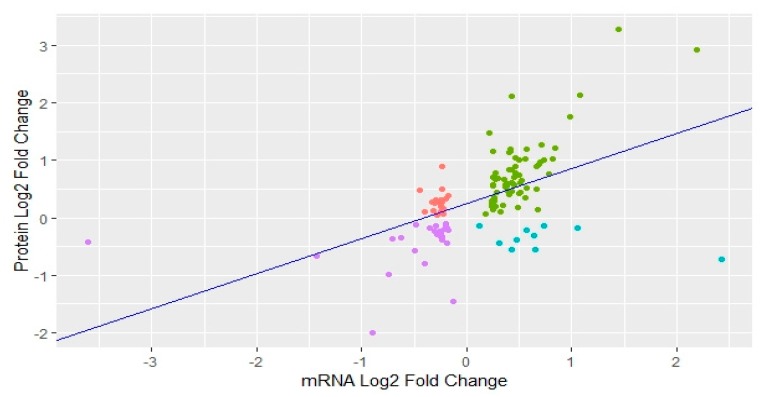
Scatter plot showing correlation between protein abundance and respective gene expression. Green: up-regulated proteins and up-regulated mRNA. Red: up-regulated proteins and down-regulated mRNA. Purple: down-regulated proteins and down-regulated mRNA. Blue: down-regulated proteins and up-regulated mRNA.

We also compared our dataset of DA proteins with the list of differentially expressed (DE) genes for the same ovarian samples from post- versus pre-pubertal heifers which was previously reported by our group [29]. Comparative analysis resulted in 126 features in common, i.e., DA proteins that were also DE genes in the previous RNA-sequencing analyses. We performed correlation and regression analysis for these 126 proteins and genes. The correlation coefficient (*r*) between expression of genes and abundance of respective proteins was positive: *r* = 0.54. In regression analysis, we regressed the fold change in differential expression of genes (independent variable) against the fold change in differential abundance of their respective proteins (dependent variable). Regression coefficient was a positive value of 0.61 while the coefficient of determination (*R*^2^) was calculated as 0.28 with *p* value < 0.0001 (Figure 5). In short, the abundance level of DA proteins was correlated to the expression of DE genes.

## 4. Discussion

This study aimed to investigate the genes and pathways involved in ovarian progesterone production—a hallmark of CL function—at the onset of puberty. To this aim, homogenized ovarian samples that represent the entirety of both ovaries were used for proteomics. Ovarian granulosa and theca cells are crucial for steroid synthesis and oocyte maturation, and so their activity is relevant to maintenance of subsequent pregnancy through progesterone signaling. After puberty (first ovulation), a CL is formed and it produces progesterone, which is necessary to maintain early pregnancy, and hence contributes to fertility [86,87]. The presence of the CL alters ovarian gene expression [88]. The identified DA proteins in post-pubertal heifers at the luteal phase compared to pre-pubertal heifers indicate their importance to CL function, as the presence of the CL cells represents the main difference between the two physiological stages. This DA proteins might have a role in puberty and subsequent fertility as they were linked in this work to essential ovarian activities. In the following sections, DA proteins are grouped and discussed according to their ovarian function.

### 4.1. Metabolic Pathways

Up-regulated DA proteins were found to be related to metabolic pathways such as glycolysis, oxidative phosphorylation, and beta-oxidation. Up-regulation of glycolysis and genes which involve glycolysis and steroidogenesis have been reported in the oocytes of post-pubertal cows when compared to pre-pubertal cows [29,89]. Oxidative phosphorylation was an enriched pathway in the list of up-regulated proteins in our results. Up-regulation of oxidative phosphorylation affects steroidogenesis through steroidogenesis acute regulatory protein (StAR) [90]. Stimulation of beta-oxidation of fatty acids for oocyte maturation through protein kinase-A signaling has also been reported in mice [91]. Up-regulation of proteins is linked to metabolic pathways in post-pubertal heifers at the luteal phase compared to pre-pubertal heifers and evidence from the cited literature suggests that these pathways play a role in progesterone synthesis. Progesterone signaling contributes to pubertal development according to the Gonodostattheory and the observation that a first short cycle is often necessary to the establishment of normal cycling in bovine species [14]. Therefore, and in the context of this study, DA proteins related to metabolic pathways might contribute to both CL function and pubertal development.

### 4.2. Transcription and Translation

We identified DA proteins that regulate transcription. Regulatory patterns of gene transcription and translation during oocyte growth, oocyte maturation, and luteinization of granulosa cells are timing specific [92,93]. Histones play a crucial role in transcriptional regulation [68]. Histone H3.3 was found to be one of the most down-regulated proteins post-puberty (fold change = −1.46). Specific methylation of H3 is associated with repression of α-inhibin, which is important for CL function and progesterone synthesis [58]. H3.3 and H4 interfere with meiosis and their depletion results in primary oocyte death and zygotic and early embryonic deaths [68]. Macro-H2A, HDGF, HP1BP3, and LMCD1 were down-regulated in post-pubertal heifers at the luteal phase in our data. These proteins are involved in the repression of transcription [94,95,96,97]. Down-regulation of specific histones and transcription repression proteins indicate enhanced transcription in post-pubertal heifers at the luteal stage compared to pre-pubertal heifers. These regulatory DA proteins probably have a role in progesterone synthesis by CL cells. These same proteins might be involved in meiotic oocyte maturation/arrest in pre-ovulatory follicles in coordination with progesterone signaling.

Increased translation and ribosomal activity, which is related to steroidogenic synthesis after LH stimulation, was observed in our data and has been seen in a previous work [98]. Some ribosomal proteins were DA in this study namely, RPL30, RPL27a, RPS8, RPS12, and RPLPO, and have also been found to be differentially expressed in mature buffalo oocytes compared to pre-pubertal oocytes [99]. Future work could use ovarian dissection to isolate granulosa cells and oocytes to investigate which cell type contributes to the significant difference observed for DA proteins. Overall, DA proteins associated with regulation of transcription and translation were found to be up-regulated, which could be explained by very active CL cells compared to the relatively less active cells in pre-pubertal ovaries.

All translation- and ribosome-related DA proteins were up-regulated in our data except for the eukaryotic initiation factor EIF4B. Translation of maternal mRNAs is regulated by secondary structures in UTRs [93,100]. EIF4B was down-regulated (FC = −1.24) in post-pubertal heifers. EIF4B stimulates EIF4A to unwind secondary structures from maternal mRNAs to enable attachment of ribosomes for translation initiation during oocyte maturation [60,101]. Time-specific translation of maternal mRNAs is necessary for oocyte maturation [60]. At luteal phase post-puberty, where no oocyte maturation is expected, the selective down-regulation of EIF4B indicates that this protein might regulates translation that is specific to oocyte maturation. Once more, isolation of oocyte gene expression would be important to confirm this idea. 

### 4.3. Complement System

The list of down-regulated proteins post-puberty was enriched for the ‘Complement and coagulation cascade pathway’. Maintenance of mature CL is dependent on lowering the immune system in CL cells [102]. Down-regulation of complement factors, which are part of the immune system, at the luteal phase in post-pubertal heifers is indicative of a mature CL being present and functional. Progesterone measurements also confirmed that post-pubertal heifers had functioning CLs in our dataset. Complement component C3 has also been identified as positively associated with oocyte maturation in pigs [103]. Another study has identified complement components C3, C4, C7, C8, C9, C-H, and C-I in human follicular fluid and associated these with oocyte maturation [104]. Differential abundance of complement components in post-pubertal heifers at the luteal phase compared to pre-pubertal heifers in our study supports the role of these proteins in CL maintenance and pubertal development. Both phenomena would have implications for subsequent fertility.

### 4.4. Extracellular Matrix Proteins

Extracellular matrix and focal adhesion proteins are important in mediating paracrine signaling in ovaries for follicular development and CL functions [105,106]. Fibronectin and asporin are examples of ECM proteins that were DA. Fibronectin was increased in abundance post-puberty, at the luteal phase. Increased fibronectin is indicative of a functioning CL [107]. Asporin (ASPN) was the most down-regulated protein in post-pubertal heifers at the luteal phase compared to pre-pubertal heifers. ASPN in theca cells is associated with growth of secondary follicles in the gonadotrophins independent stage [57]. The up-regulation of fibronectin and the down-regulation of asporin both tell a story of a functioning CL and reduced follicular growth, which could be expected by the presence of CL cells at the luteal phase which were not present before.

Some ECM proteins, such as SERPINs and Tenascin-X (TNXB), might be involved in oocyte maturation. Nine SERPIN proteins, including SERPINE2, were down-regulated in post-pubertal heifers compared to pre-pubertal heifers. ECM proteins, including SERPINs and ITIH1, are found to be specific to oocyte maturation and SERPINE2 has been proposed as a biomarker for oocyte maturation [108,109]. Tenascin-X (TNXB) was among the top down-regulated proteins (FC = −1.12) in post-pubertal heifers in our results. Transcription of the TNXB gene is increased in pre-ovulatory follicles and pregnancy competent oocytes [64,110]. As we did not separate specific cell types in our study, the role of these nine SERPINs and TNXB in oocyte maturation requires validation. 

Agrin (AGRN), lamin-A (LMNA), lamin-B (LMNB), laminin, clusterin (CLU1) and vimentin (VIM) are also ECM proteins that were down-regulated in our data. Remodeling of extracellular matrix proteins and adhesion proteins has also been reported in the leutinization of cells that form the CL. Proteins such as integrin-vα and laminin are associated with leutinization [111]. Lamins and laminins are part of the basal lamina that separates granulosa cells from theca cells in pre-ovulatory follicles. They are important ECM proteins that mediate the interactions between granulosa cells, the thecal layer, and the oocyte. This basal lamina degrades during CL formation [107]. Decreased abundance of lamins in our study agrees with the presence of functioning CL cells post-puberty, a major contrast to the pre-pubertal samples. Lamins are increased in expression during meiotic oocyte maturation [112]. Laminin A and B have increased expression levels associated with oocyte maturation in mice [113]. The reverse, down-regulation, is expected in the luteal phase. Similarly, CLU1 was down-regulated in our data, which could be expected for the luteal phase since increased levels of CLU1 have been related to oocyte maturation in bovine previously [56]. Differential abundance of ECM proteins in post-pubertal as compared to pre-pubertal heifers probably relates to CL formation and function, with implications for pubertal development and subsequent fertility.

### 4.5. Steroidogenesis

The up-regulation of steroidogenic proteins in our study can probably be explained by the presence of functioning CL cells post-puberty which were absent pre-puberty. Steroidogenesis starts with the precursor acetyl-CoA, which leads to synthesis of cholesterol and finally steroid hormones. We observed 32 DA proteins between pre- and post-pubertal Brahman heifers that are relevant to steroidogenesis. Highlighting these DA proteins, we were able to annotate the steroidogenesis pathway in *Bos indicus* animals.

Acetyl-CoA results from different metabolic pathways, including glycolysis. Nine up-regulated proteins from our results play a role in glycolysis and result in pyruvate synthesis [114], which produces acetyl-CoA in a subsequent reaction [115]. Acetyl-CoA is converted to citrate by citrate synthase (CS). Citrate can enter the tricarboxylic acid cycle or be transported to cytosol by citrate transport protein (CTP). Citrate is cleaved and converted into acetyl-CoA and oxaloacetate by ATP citrate lyase (ACL) [49]. Acetyl-CoA can be used to synthesize fatty acids or cholesterol or to enter into thetricaboxylic acid cycle [115,116]. The above-mentioned proteins CS, CTP, and ACL were up-regulated in our data. Up-regulation of proteins in glycolysis and subsequent steps indicates their important role in contributing acetyl-CoA as a precursor for up-regulation of steroidogenesis at puberty. 

Bothde novo synthesis and beta-oxidation of fatty acids were observed. Fatty acid synthase (FASN) was up-regulated post-puberty. FASN converts acetyl-CoA to palmitate, the most abundant saturated fatty acid [48]. Enzymes acyl-CoA dehydrogenase (ACAD) and a tri-functional protein (HADHA) were up-regulated post-puberty. These enzymes, through beta-oxidation, contribute acetyl-CoA to steroidogenesis [41,117]. The enzymes involved in branched chain amino degradation, including acetyl-CoA acetyltransferase-1 (ACAT1), 3-hydroxymethylglutaryl-CoA synthase-1 (HMGCS1), hydroxysteroid-17-beta dehydrogenase-10 (HSD17B10), and acyl-CoA dehydrogenase (ACAD8) were up-regulated post-puberty. Branched chain amino-acid degradation also produces acetyl-CoA [118]. Increased abundance of DA proteins belonging to fatty acid synthesis, beta-oxidation, and branched chain amino-acid degradation suggests increased availability of acetyl-CoA for steroidogenesis in the luteal phase post-puberty.

Different enzymes converting acetyl-CoA to cholesterol, including acetyl-CoA acetyl transferase2 (ACAT2), HMG-CoA synthase, lanosterol synthase (LSS), 24-dehydrocholesterol reductase (DHCR24), and glucose 6 phosphate dehydrogenase (G6PDH) were also up-regulated post-puberty. ACAT2 converts acetyl-CoA to acetoacetyl-CoA, which is further converted to hydromethylglutaryl-CoAby HMG-CoA synthase [44]. HMG-CoA in further reactions is converted to oxidosqualene, which is converted to lanosterol by LSS [45]. DHCR24 converts desmosterol to cholesterol and all reducing reactions during cholesterol synthesis are catalyzed by G6PDH [41,119]. Therefore, these up-regulated proteins contribute to synthesis of cholesterol in the pathway upstream to steroid hormone production.

The apolipoproteins A-I, A-II, and C-III were down-regulated post-puberty. Apolipoproteins sustain cholesterol homeostasis in cells, removing it when it becomes excessive to avoid cellular cholesterol toxicity [120,121,122]. Up-regulation of enzymes that contribute to cholesterol synthesis and down-regulation of cholesterol efflux proteins together indicates increased supply of cholesterol for steroidogenesis post-puberty. 

Cholesterol side-chain cleavage enzyme (CYP11A1), ferredoxin (FDX), ferredoxin reductase (FDXR), and 3-beta-hydroxysteroid dehydrogenase (HSD3B1) were up-regulated while steroid 17-alpha-hydroxylase (CYP17A1) was down-regulated in our results. Conversion of cholesterol to pregnenolon is mediated by CYP11A1. FDXR and FDX help in electron transfer for this reaction. Pregnenolon is converted to progesterone by 3-beta-hydroxysteroid dehydrogenase (HSD3B1) [42,43]. CYP17A1 converts progesterone to androstenedione [123], which in further reactions is converted into estrogen [124]. Up-regulation of progesterone synthesis proteins and down-regulation of estrogen-related proteins indicate high levels of progesterone production, which is typical of the luteal phase. 

The process of steroidogenesis is the same for both progesterone and estrogen up to the synthesis of cholesterol. The up-regulation of this process up to cholesterol synthesis may be important for both the estrogenic and the luteal phase post-puberty. To confirm the overall relevance of these DA proteins to the onset of puberty, future research could compare pre-pubertal heifers to post-pubertal heifers in all phases of the estrus cycle.

### 4.6. Progesterone Signaling Regulating Puberty and Fertility

Progesterone and its membrane receptors are involved in oocyte maturation in the pre-ovulatory follicle, in fertilization processes, and in the maintenance and development of the pregnancy [125,126,127]. These phenomena, which are affected by progesterone signaling, contribute to overall female fertility. Progesterone signaling is complex and some of the same pathways are relevant to mature CL cells and to oocyte maturation. The DA proteins G inhibitory protein GNAI2, adenylyl cyclase associated protein (CAP-1), adenylate kinase (ADK), and protein kinase A regulatory subunit II α and β (PRKAR2α and PRKAR2β) all contribute to activate the transcription factor cAMP responsive regulatory element binding protein (CREB). In turn, CREB regulates the transcription of proteins related to CL functioning [128,129]. The fact that these proteins are DA herein might be explained by the presence of the CL being unique to post-pubertal heifers.

Some of these DA proteins are also known for their roles in progesterone signaling related to oocyte maturation: GNAI2, CAP-1, and ADK are, for example. GNAI2 was down-regulated whileCAP-1 and ADK were up-regulated in post-pubertal heifers compared to pre-pubertal heifers. These proteins are involved in progesterone-mediated oocyte maturation/arrest. Progesterone through membrane progesterone receptors activates GNAI2, which inhibits adenylyl cyclase (AC) to suppress levels of cAMP in oocytes [24,130,131,132,133,134]. Similarly, CAP-1 and ADK are also involved in the regulation of cAMP levels [135,136]. Suppression of cAMP relieves oocytes from meiotic arrest and hence causes oocyte maturation [130,132,133,137,138]. PRKAR2α and PRKAR2β were also down-regulated in post-pubertal heifers compared to pre-pubertal heifers. Interestingly, increased levels of PRKAR2α and PRKAR2β are associated with progesterone-, mediated oocyte maturation [24,131]. 

Tyrosine monoxygenase activation proteins YWHAG, YWHAE and YWHAZ were up-regulated in post-pubertal heifers compared to pre-pubertal heifers. These proteins suggestively bind to Cdc25 and prevent its binding to meiosis promoting factor (MPF) [24,139,140,141]. As a result, oocyte maturation is prevented when YWHAG, YWHAE, and YWHAZ are up-regulated. 

Granulosa cells contribute to oocyte maturation at the pre-ovulatory stage and then contribute to the maintenance of pregnancy after being transformed into luteal cells. Both of these phenomena are associated with progesterone signaling at two different stages of the estrous cycle. Low levels of progesterone (and other factors) produced by granulosa cells facilitate follicular growth and oocyte maturation. High levels of progesterone produced by the CL might contribute to oocyte arrest during the luteal phase. The differential abundance of these progesterone-signaling proteins between ovarian tissues of pre-pubertal heifers and post-pubertal heifers at the luteal phase indicates that these proteins are probably differentially regulated in luteinized granulosa cells in the CL compared to non-luteinized granulosa cells. 

### 4.7. Retinoic Acid Signaling

Our results revealed increased abundance of retinal dehydrogenase (RALHD-1), protein phosphatase-2A (PPP2R1A), and cellular retinoic acid binding protein (CRABP1). These proteins are relevant to retinoic acid (RA) signaling. Retinoic acid is involved in stimulation of progesterone synthesis in the CL [142]. Also, the direct genomic action of RA signaling or its regulation of other effector pathways aids oocyte maturation [143,144]. CRABP1 protein is differentially expressed in different stages of bovine oocyte maturation [56]. It inhibits RA activity and also stimulates PPP2R1A via ERK1/2 kinases to delay the cell cycle [144,145]. Cell cycle regulation is probably also important to the formation of the CL via cellular differentiation and proliferation processes, as discussed above. 

### 4.8. TGF-β Signaling in Regulation of Oocyte Maturation/Arrest

Follistatin (FST), ASPN, biglycan (BGN), decorin (DCN), TGF-β-induced protein (beta-ig-h3/TGFBIp) and TGF-β1-induced transcript-1 protein (TGFBI1/Hic-5) were decreased in abundance in post-pubertal heifers compared to pre-pubertal heifers. FST, ASPN, and DCN inhibit TGF-β superfamily members [146,147,148,149]. TGF-β superfamily members have roles in CL progesterone secretion, follicular growth, and oocyte maturation [22,150,151]. TGF-β1 is also involved in regression of the CL and its increased levels are associated with decrease in progesterone production [152]. TGF-β1 inhibits LH receptors and allows completion of oocyte cytoplasmic maturation [153,154]. Support from cited literature and differential abundance of TGF-β-related proteins suggest that TGF-β signaling is important for CL function post-puberty.

### 4.9. Pleiotrophin and Progesterone Sinaling on Focal Adhesion, Cytoskeleton, and Microtubules

We suggest a putative role of pleiotrophin in coordination with focal adhesion and cytoskeleton proteins at the luteal phase in post-pubertal heifers. Cytoskeleton and microtubules play a role in progesterone synthesis and oocyte maturation [155,156,157,158,159]. Pleiotrophin, ITGAv (an integrin), and ADD-1 were up-regulated, while filamin (FLNA) and actinin (ACTN1) were down-regulated in post-pubertal heifers at the luteal phase. Pleiotrophin signaling through ITGAvβ3 (heterodimer of ITGAv and ITGAβ) has been reported [160]. ITGAvβ3is involved in phosphorylation of cytoskeleton proteins and focal adhesion proteins, including FLNA and ACTN1 [161,162]. Signaling through integrins down-regulates cytoskeleton structures in cells [162]. Adducin heterodimers, composed of β-adducin (ADD-2) and α-adducin (ADD-1), regulate the actin-spectrin complex in the cytoskeleton [163]. Phosphorylation of ADD-2 by pleiotrophin signaling results in cytoskeletal dissociation [164]. Tubulin-α, tubulin-β subunits, microtubules-associated protein (MAP), dynein light and heavy chains (DYN), and dynactin (DCTN-2) were up-regulated in post-pubertal heifers compared to pre-pubertal heifers in our results. These proteins are involved in microtubule and spindle fiber assembly and function [165]. Dissociation of the cytoskeleton and microtubules is associated with more progesterone synthesis in granulosa cells [159]. Nuclear mitotic apparatus-1 (NuMA-1) was down-regulated in post-pubertal heifers. NuMA organizes microtubules at spindle poles in coordination with DYN and DCTN [166] and plays an important role in meiotic oocyte maturation by organizing tubulins [167]. Similarly, specific hyper-phosphorylation of ADD-1 causes abnormal assembly of spindle fibers in meiosis and mitosis [168,169]. Mitosis-associated proteins are very important in the context of forming the CL since the ceasing of cell division is required for cell differentiation and growth processes that transform granulosa cells into luteal cells [16]. Progesterone, through its membrane receptors, is involved in spindle fiber assembly arrangement at different stages of meiosis during oocyte maturation [126] and during mitosis of granulosa cells [170,171]. Differential abundance of proteins related to the cytoskeleton, spindle fibers, and cell division, corroborated by the cited literature, are evidence for the involvement of these proteins in the formation of the CL.

### 4.10. Association of DA Proteins with Reproductive Traits

Genes of 10 DA proteins from our study were associated with important reproductive traits according to the animal QTL database [40]. The DA protein ezrin (EZR) was associated with “length of reproductive life” and “calving ease” in cattle [79]. Functionally, ezrin is involved in cytoskeleton arrangement [172]. T-complex protein component-1 (TCP-1) and T-complex protein component-8 (CCT-8) were DA in our data and were associated with “conception rate” in cattle [82]. Both of these chaperone proteins help in folding actin and tubulin in cytoskeleton and spindle formation [173,174]. Calpain-1 (CAPN1) was another DA protein in our data that has been previously associated with two reproductive traits, namely “post-partum anestrous interval” and “IGF-1 levels” [83,175]. CAPN1 seems to play a role in cumulus cell-oocyte complex expansion and oocyte maturation [176] and from our data it seems it might also be involved in CL function. Leucine aminopeptidase (LAP3) is a DA protein associated with “calving ease” in cattle [84]. LAP3 expression increases in theca cells of mature pre-ovulatory oocytes in humans in the presence of high levels of progesterone. It has also been reported to play a role in oocyte maturation in *Haemaphysalislongicornis* tick [177,178]. FST, another DA protein, has been associated with “conception rate” [81]. Increased FST mRNA levels have been observed in mature oocytes, while decreased levels have been associated with oocyte arrest in cattle [179]. Serpine-2 is associated with fertilization rates in cattle [85]. It has been found to be differentially expressed in the granulosa cells of bovine ovaries [66]. Association of these DA proteins with reproductive trait QTLs seems to support their involvement with female fertility, which could be expected for proteins that are involved in the formation and function of the CL.

## 5. Conclusions

This study has provided a set of ovarian proteins that were found to have altered abundance levels as a result of CL activity, progesterone signaling, and puberty in *Bos indicus* heifers. Selective regulation of proteins involved in various metabolic pathways indicated their role in up-regulating overall steroidogenesis upon puberty. The reported proteins have enhanced our current understanding of pathways that are regulated in CL cells and therefore contribute to progesterone signaling, with implications for oocyte maturation, puberty, and subsequent fertility. Some DA proteins are coded by genes in QTLs for female reproductive traits and can be further scrutinized in the search for causative mutations.

## Figures and Tables

**Table 1:** Enriched pathways for up- and down-regulated proteins in post-pubertal heifers at the luteal phase compared to pre-pubertal heifers. Legend: ECM, extracellular matrix.

Enriched Pathways	Total Proteins	DA Benjamini *p* Value
Up-Regulated Proteins		
Ribosome	35	2.8 × 10 ^−5^
Metabolic pathways	81	9.5 × 10 ^−4^
Oxidative phosphorylation	24	8.4 × 10 ^−4^
Down-Regulated Proteins		
Complement and coagulation cascade	18	31.4 × 10 ^−6^
Systemic lupus erythematosus	17	5.2 × 10 ^−7^
Focal adhesion	22	5.2 × 10^−4^
ECM receptor interaction	15	7.6 × 10^−4^
Alcoholism	15	4.4 × 10^−4^

**Table 2:** Numbers of up- and down-regulation of proteins in pathways in post-pubertal heifers at the luteal phase compared to pre-pubertal heifers.

Pathways	DA Proteins	Up-Regulated	Down-Regulated
Glycolysis	9	9	0
TCA cycle	12	12	0
Pentose phosphate pathway	7	7	0
Oxidative phosphorylation	24	24	0
Fatty acid metabolism	9	9	0
Branched chain amino-acid degradation	11	10	1
Terpeniod backbone biosynthesis	4	4	0
Cholesterol metabolism	10	8	2
Cholesterol efflux	3	0	3
Ovarian steroidogenesis	15	11	4
Oocyte maturation and oocyte meiosis	12	8	4
cAMP signaling	6	2	4
Estrogen signaling	8	5	3
Cell division	6	3	3
Microtubule processing	4	4	0
Regulation of actin cytoskeleton	20	10	10
Focal adhesion	28	5	23
Cell matrix adhesion	5	0	5
ECM receptor interaction	17	2	15
Protein processing	21	20	1
Ribosome	35	35	0
Translation	38	37	1
Nucleosome assembly	15	1	14
Regulation of transcription	11	5	6
Glutathione metabolism	11	7	4
Cell redox homeostasis	13	13	0

TCA: Tricarboxylic Acid Cycle. cAMP: Cyclic Adenosine Monophosphate.

**Table 3:** Top twenty up- and down-regulated proteins from the current study and their function according to the cited literature, in the context of puberty and fertility.

Proteins	log_2_ Fold Change	Cellular Process	Puberty-Related Function	Reference
DHCR24	3.28	Cholesterol synthesis	Steroidogenesis	Robert K. Murray et al. [41]
FDX1 ADX	3.02	Electron transfer	Steroidogenesis	Miller et al. [42]
HSD3B	2.92	Progesterone synthesis	Steroidogenesis	Miller et al. [43]
HMGCS	2.91	Cholesterol synthesis	Steroidogenesis	Russell et al. [44]
LSS	2.87	Lanosterol synthesis	Steroidogenesis	Christianson et al. [45]
FDXR	2.66	Electron transfer	Steroidogenesis	Miller et al. [42]
TPD52	2.66	Cell cycle regulation	Scrotal circumference association	Meirelles et al. [46]
QPRT	2.13	NAD+ biosynthesis	Antioxidation in steroidogenesis	Aguilera-Méndez, Fernández-Lainez et al. [47]
FASN	2.11	Fatty acid synthesis	Up-regulation of steroidogenesis	Wakil et al. [48]
CYP11A1	2.11	Pregnenolone synthesis	Up-regulation of steroidogenesis	Miller et al. [43]
ACLY	1.75	Citrate synthesis	Up-regulation of steroidogenesis	Guay, Madiraju et al. [49]
IDH3A	1.65	TCA cycle	Up-regulated in old age oocytes	Itami, Kawahara-Miki et al. [50]
Uncharacterized	1.48			
NNT	1.37	Electron transport chain	Antioxidant in steroidogenesis	Roucher-Boulez, Mallet-Motak et al. [51]
NDUFC2	1.30	Electron transport chain	Antioxidant in oocyte maturation	Payton, Rispoli et al. [52]
ACAT2	1.29	Acetyl-CoA metabolism	Up-regulation of steroidogenesis	Russell et al. [44]
LONP1	1.26	Mitochondrial activity	Up-regulation of steroidogenesis	Rone, Midzak et al. [53]
AIFM1	1.26	Pro-apoptotic activity	Atresia of antral follicles	Craig, Singh et al. [54]
PTN	1.21	Focal adhesion	Female infertility	Muramatsu, Zou et al. [55]
RBP1	1.20	Retinoic acid signaling	Oocyte maturation	Salhab, Tosca et al. [56]
ASPN	−1.99	Extracellular matrix	Associated with secondary follicle growth Transcription for CL functioning	Aoyama, Shiraishi et al. [57] Meldi, Gaconnet et al. [58]
H3F3A/B	−1.46	Nucleosome assembly	Chromatin remodeling in oocyte maturation	Fournier, Dufort et al. [59]
EIF4B	−1.24	Translation	Translation in oocyte maturation	Ellederová, Cais et al. [60]
SERPINA3	−1.23	Extra cellular matrix	Decreased abundance in 11-days CL Oocyte competence associated	Zalman, Ireland et al. [61] Hamel, Dufort et al. [62]
Uncharacterized PTI	−1.15			
TN-X	−1.12	Extracellular matrix	Marker for CL function. Up-regulated in antral follicles and competent oocytes	Dominguez, Cho [63] Cibelli, Iager et al. [64]
ApoC3	−1.09	Cholesterol efflux	Steroidogenesis, down-regulated in cumulus cells of mature oocytes	Shao, Chian et al. [65]
SERPINE2	−1.01	Extracellular matrix	Differential expression in antral follicle and mature oocytes	Bédard, Brûlé et al. [66]
SERPINA3.3	−1.00	Extracellular matrix	Expressed in follicular fluid during oocyte maturation	Ducolomb, González-Márquez et al. [67]
Uncharacterized GSTM	−0.99			
H2AFY2	−0.98	Nucleosome assembly	Repressed transcription on meiosis	Wang, Xu et al. [68]
LMCD1	−0.96	Regulation of transcription	Regulation of transcription in spermatogenesis	Griffin, Dunmore et al. [69]
BLT	−0.93	Proteolytic activity	Second maturation of oocyte	Yamane et al. [70]
VCAN	−0.90	Extracellular matrix	Oocyte maturation quality	Dunning, Watson et al. [71]
PRELP	−0.82	Extracellular matrix	Follicular development	Irving-Rodgers and Rodgers et al. [72]
LAMB2	−0.82	Extracellular matrix	Decreases after oocyte maturation	Budna, Celichowski et al. [73]
H1F0	−0.82	Nucleosome assembly	Oocyte maturation	Niu, Zi et al. [74]
FST	−0.82	TGF-β signaling	Progesterone synthesis by suppressing TGF-β signaling Oocyte maturation	Kayani, Glister et al. [75] Adona, Leal et al. [76]
DDX17	−0.80	Transcription	Differential expression in cumulus cells	Assou, Haouzi et al. [77]
FBN1	−0.78	Extracellular matrix	Oocyte quality marker	Powell, Manandhar at al. [78]

**Table 4:** Ten genes that code for differentially abundant proteins in the current experiment mapped to genomic regions associated with reproductive traits in cattle.

No.	Gene	Chromosome/Locus (Mbp)	Traits Associated	Reference
1	*NNT*	20 (31.2)	Length of reproductive life	Kolbehdari, Wang et al. [79]
2	*ACAT2*	9 (97.5)	Length of reproductive life Conception rate Daughter pregnancy rate	Dikmen, Wang et al. [80]
3	*TXN*	5 (75.3)	Daughter pregnancy rate	Ortega, Denicol et al. [81]
4	*EZR*	9 (96.6)	Length of reproductive life Calving ease	Kolbehdari, Wang et al. [79]
5	*TCP1*	1 (6.5)	Conception rate	Cochran, Cole et al. [82]
6	*CCT8*	1 (6.5)	Conception rate	Cochran, Cole et al. [82]
7	*CAPN1*	29 (32.5–34.5) 29 (32.5–34.5)	Post-partum anestrus interval (PPAI) IGF-1 levels	Collis, Fortes et al. [83] Collis, Fortes et al. [83]
8	*LAP3*	6 (38.6)	Calving ease	Bongiorni, Mancini et al. [84]
9	*FST*	20 (25.6)	Conception rate	Ortega, Denicol et al. [81]
10	*SERPINE2*	2 (112.9)	Fertilization rate	Cochran, Cole et al. [85]

## Supplementary Materials

The following are available online at https://www.mdpi.com/2073-4425/10/11/923/s1, Table S1: Total differentially expressed proteins in pre- versus post-pubertal Brahman heifers, Table S2: Pathways and differentially abundant proteins in post-pubertal heifers compared to pre-pubertal heifers involved in ovarian activities at puberty.
