# Supplementary material for: Proteomics Recapitulates Ovarian Proteins Relevant to Puberty and Fertility in Brahman Heifers (Bos indicus L.)
Source: Genes (Basel). 2019 Nov 12;10(11):923. doi: 10.3390/genes10110923 (PMC6895798; doi:10.3390/genes10110923)
Supplement: Supplementary file 1 [file genes-10-00923-s001.zip › Table S1.pdf]

Table S1: Total differentially expressed proteins in pre- vs post-pubertal Brahman heifers.				
**ProteinID***	**Gene**	**log2FC***	**adj.pvalue**	**Protein Names**
A6QR14	DHCR24	3.28	0.000	24-dehydrocholesterol reductase
P00257	FDX1 ADX	3.02	0.000	Adrenodoxin
P14893	HSD3B	2.92	0.000	3 beta-hydroxysteroid dehydrogenase
Q3ZC79	HMGCS1	2.91	0.000	3-hydroxy-3-methylglutaryl coenzyme A synthase
F1MTJ9	LSS	2.87	0.001	Terpene cyclase
Q148K8	FDXR	2.66	0.000	NADPH:adrenodoxin oxidoreductase
Q3ZCA8	TPD52	2.66	0.000	Tumor protein D52
F1MHB8	QPRT	2.13	0.000	Nicotinate-nucleotide pyrophosphorylase
F1N647	FASN	2.11	0.000	Fatty acid synthase
A3KMX9	CYP11A1	2.11	0.000	Cholesterol side-chain cleavage enzyme
Q32PF2	ACLY	1.75	0.000	ATP-citrate synthase
P41563	IDH3A	1.65	0.000	Isocitrate dehydrogenase
F1N6P2		1.48	0.000	Uncharacterized protein
P11024	NNT	1.37	0.000	NAD(P) transhydrogenase
Q02827	NDUFC2	1.30	0.000	NADH dehydrogenase [ubiquinone] 1 subunit C2
Q1JPB6	ACAT2	1.29	0.000	Acetyl-Coenzyme A acetyltransferase 2
Q59HJ6	LONP1	1.26	0.000	Lon protease homolog, mitochondrial
E1BJA2	AIFM1	1.26	0.000	Apoptosis inducing factor mitochondria associated
P21782	PTN	1.21	0.000	Pleiotrophin (PTN)
P02694	RBP1	1.20	0.000	Retinol-binding protein 1
F1MUZ9	HSPD1	1.19	0.000	60 kDa heat shock protein, mitochondrial
Q29RK1	CS	1.19	0.000	Citrate synthase, mitochondrial
P41976	SOD2	1.18	0.000	Superoxide dismutase
P79110	SLC25A1	1.17	0.000	Tricarboxylate transport protein
P12234	SLC25A3	1.16	0.000	Phosphate carrier protein, mitochondrial
Q3SZ54	EIF4A1	1.15	0.000	Eukaryotic initiation factor 4A-I
Q148D3	FH	1.15	0.000	Fumarate hydratase
P61603	HSPE1	1.14	0.000	10 kDa heat shock protein
V6F7X8	REXO2	1.10	0.000	Oligoribonuclease
P13272	UQCRFS1	1.09	0.000	Cytochrome b-c1 complex subunit Rieske
P49410	TUFM	1.09	0.000	Elongation factor Tu,
P12344	GOT2	1.04	0.000	Aspartate aminotransferase
F1MMK2	G6PD	1.03	0.000	Glucose-6-phosphate 1-dehydrogenase
Q3ZCH0	HSPA9	1.03	0.000	Stress-70 protein
E1BA13		1.02	0.000	Uncharacterized protein
P00125	CYC1	1.02	0.000	Cytochrome c1, heme protein
Q01321	NDUFA4	1.00	0.000	Cytochrome c oxidase subunit
Q0VCM4	PYGL	1.00	0.000	Glycogen phosphorylase, liver form
F1MZT4	MX2	0.99	0.000	Interferon-induced GTP-binding protein
Q32LG3	MDH2	0.98	0.000	Malate dehydrogenase, mitochondrial
Q862H7	S100A11	0.97	0.000	Protein S100 (S100 calcium-binding protein)
P35705	PRDX3	0.97	0.000	Thioredoxin-dependent peroxide reductase
G3N3D4	KCTD12	0.95	0.000	Potassium channel tetramerization domain protein
F1MMR6	CTSD	0.92	0.000	Cathepsin D
P68002	VDAC2	0.92	0.000	Voltage-dependent anion-selective channel protein
P00423	COX4I1	0.90	0.000	Cytochrome c oxidase subunit 4 isoform 1
P11179	DLST	0.90	0.000	Dihydrolipoyllysine-residue succinyltransferase
F1N0I2	NR2C1	0.89	0.000	Nuclear receptor subfamily 2 group C member 1

P48644	ALDH1A1	0.89	0.000	Retinal dehydrogenase 1
Q9TVD2	IDH	0.88	0.001	NAD(+)-isocitrate dehydrogenase subunit 1
P05630	ATP5F1D	0.87	0.000	ATP synthase subunit delta, mitochondrial
Q2NL19	IMMT	0.86	0.000	MICOS complex subunit MIC60 (Mitofilin)
P01966	HBA	0.86	0.000	Hemoglobin subunit alpha
A1A4N9	NME1	0.86	0.000	Nucleoside diphosphate kinase
Q9XSG3	IDH1 ICDH	0.85	0.000	Isocitrate dehydrogenase [NADP] cytoplasmic
Q3SZ81	PSMC3	0.84	0.000	PSMC3 protein (Fragment)
P08166	AK2	0.84	0.000	Adenylate kinase 2, mitochondrial (AK 2)
P20004	ACO2	0.84	0.000	Aconitate hydratase, mitochondrial (Aconitase)
P42028	NDUFS8	0.83	0.000	NADH dehydrogenase
Q862L1		0.83	0.000	Similar to tumor protein
P45879	VDAC1	0.80	0.000	Voltage-dependent anion-selective channel protein
Q3ZBF6	ACADS	0.80	0.000	Short-chain specific acyl-CoA dehydrogenase
Q1LZ72	CNPY2	0.80	0.000	Canopy 2 homolog (Zebrafish)
P00126	UQCRH	0.79	0.000	Cytochrome b-c1 complex subunit 6
Q3T145	MDH1	0.78	0.000	Malate dehydrogenase, cytoplasmic
Q2KJ39	RCN3	0.78	0.000	Reticulocalbin-3
Q3MHX5	SUCLG2	0.78	0.000	Succinate--CoA ligase [GDP-forming] subunit beta
Q3T0L7	RPL28	0.78	0.000	60S ribosomal protein L28
Q0QEQ4	IDH1	0.77	0.000	Isocitrate dehydrogenase 1 (Fragment)
P32007	SLC25A6	0.77	0.000	ADP/ATP translocase 3
Q3MHW4	SQRDL	0.77	0.000	SQRDL protein
Q5E9F5	TAGLN2	0.76	0.000	Transgelin-2
P00429	COX6B1	0.76	0.000	Cytochrome c oxidase subunit 6B1
Q3ZCI4	PGD	0.74	0.000	6-phosphogluconate dehydrogenase
Q76LV1	HSP90AB1	0.73	0.000	Heat shock protein HSP 90-beta
Q28035	GSTA1	0.72	0.000	Glutathione S-transferase A1
Q3MI02	UQCRC1	0.70	0.000	UQCRC1 protein (Fragment)
P02721	ATP5PF	0.70	0.000	ATP synthase-coupling factor 6, mitochondrial
F1MH57	ACAD8	0.70	0.000	Isobutyryl-CoA dehydrogenase, mitochondrial
G3N3W3	SLC25A5	0.70	0.000	ADP/ATP translocase 2
F1MWK6	MFAP2	0.70	0.000	Microfibrillar-associated protein 2
P00829	ATP5F1B	0.70	0.000	ATP synthase subunit beta, mitochondrial
Q7JAT2	ATP8 atp8	0.69	0.000	ATP synthase protein 8
E2GEZ1	Tom22	0.69	0.000	Translocase of outer mitochondrial membrane22
A6H7J6	P4HB	0.69	0.000	Protein disulfide-isomerase
A7Z066	CANX canx	0.69	0.000	Calnexin (Canx protein)
F1MLB8	ATP5F1A	0.68	0.000	ATP synthase subunit alpha
F1MWR3	ETFA	0.68	0.000	Electron transfer flavoprotein subunit alpha
P13621	ATP5PO	0.67	0.000	ATP synthase subunit O, mitochondrial
P13620	ATP5PD	0.67	0.000	ATP synthase subunit d, mitochondrial
P00426	COX5A	0.67	0.000	Cytochrome c oxidase subunit 5A
Q58DR8	SUCLG1	0.67	0.000	Succinate--CoA ligase [ADP/GDP-forming] subunit
Q2TBV3	ETFB	0.66	0.000	Electron transfer flavoprotein subunit beta
Q3SZE6	NDUFA10	0.65	0.001	NDUFA10 protein
P33097	GOT1	0.65	0.000	Aspartate aminotransferase, cytoplasmic
D4QBB4	HBB GLNA1	0.65	0.000	Globin A1 (Hemoglobin beta)
Q3ZBD1	RAB1A	0.63	0.000	RAB1A, member RAS oncogene family
P23004	UQCRC2	0.61	0.000	Cytochrome b-c1 complex subunit 2,

J9ZXG5		0.60	0.000	Integrin alpha V subunit
P07107	DBI	0.60	0.000	Acyl-CoA-binding protein (ACBP)
Q3ZC41	ACAA1	0.60	0.000	Acetyl-CoA acyltransferase 1
F1ME65	CKAP4	0.60	0.000	Cytoskeleton associated protein 4
Q28851	ATP5MF	0.60	0.000	ATP synthase subunit f, mitochondrial
P10096	GAPDH	0.59	0.000	Glyceraldehyde-3-phosphate dehydrogenase
Q3ZCD7	TECR	0.58	0.000	Very-long-chain enoyl-CoA reductase
O02691	HSD17B10	0.58	0.000	3-hydroxyacyl-CoA dehydrogenase type-2
Q5E936	TXNDC12	0.58	0.000	Thioredoxin domain-containing protein 12
Q9GMB8	SARS SERS	0.57	0.000	Serine--tRNA ligase, cytoplasmic
G3X757	VCP	0.57	0.000	Transitional endoplasmic reticulum ATPase
F1MDC1	RRBP1	0.57	0.000	Uncharacterized protein
P05631	ATP5F1C	0.56	0.000	ATP synthase subunit gamma
Q3SZ00	HADHA	0.56	0.000	HADHA,Trifunctional enzyme subunit alpha
Q6VBM2	PAICS	0.56	0.000	Phosphoribosylaminoimidazole carboxylase
F1MIH9	PLBD2	0.55	0.000	Phospholipase B-like
Q3T0Q4	NME2	0.54	0.000	Nucleoside diphosphate kinase B
P38447	ENDOG	0.54	0.021	Endonuclease G, mitochondrial
A5D7S0	NAPA	0.54	0.000	Alpha-soluble NSF attachment protein
A5D973	PPP2R1A	0.53	0.004	Alpha isoform of regulatory subunit A
P00432	CAT	0.52	0.000	Catalase
Q5E9X4	LRRC59	0.51	0.000	Leucine-rich repeat-containing protein 59
G8JKZ8	TXN	0.51	0.000	Thioredoxin
A5D9E7	HADHB	0.51	0.000	Mitochondrial trifunctional protein, beta subunit
Q3SYU2	EEF2	0.51	0.000	Elongation factor 2 (EF-2)
D4QBB3	HBB	0.51	0.000	Hemoglobin beta
F1MMP5	ITIH1	0.50	0.000	Inter-alpha-trypsin inhibitor heavy chain H1
P31976	EZR VIL2	0.49	0.000	Ezrin (Cytovillin)
Q2KJH6	SERPINH1	0.49	0.000	Serpin H1
Q2KJC5	HADH	0.49	0.000	Hydroxyacyl-Coenzyme A dehydrogenase
E1BII3		0.49	0.000	Uncharacterized protein
F1MBQ0	DYNC1LI2	0.48	0.000	Dynein cytoplasmic 1
G3MXE1		0.48	0.000	Uncharacterized protein
F1MBC7		0.47	0.000	Cytochrome c oxidase subunit 6A
Q8M444	COX2	0.47	0.000	Cytochrome c oxidase subunit 2
A6QLL8	ALDOA	0.47	0.000	Fructose-bisphosphate aldolase
P61356	RPL27	0.46	0.000	60S ribosomal protein L27
Q5E9M1	ARL6IP5	0.46	0.000	PRA1 family protein 3
L7R5X3	eef1d	0.45	0.000	Eukaryotic translation elongation factor 1 delta
G5E5A9	FN1	0.45	0.000	Fibronectin
F1MJQ1	RAB7A	0.45	0.000	Ras-related protein Rab-7a
Q7M2Y2		0.44	0.000	Tumor-specific transplantation antigen
Q3ZBH0	CCT2	0.44	0.000	T-complex protein 1 subunit beta
P00435	GPX1	0.44	0.000	Glutathione peroxidase 1
A4FUA8	CAPZA1	0.44	0.010	F-actin-capping protein subunit alpha-1
P68103	EEF1A1	0.44	0.000	Elongation factor 1-alpha 1
F1MB08	ENO1	0.43	0.000	Alpha-enolase
P23805	CGN1	0.43	0.000	Conglutinin
Q56JV7		0.43	0.000	40S ribosomal protein S30
Q3ZC87	PKM2	0.43	0.000	Pyruvate kinase

O46415	FTL	0.42	0.000	Ferritin light chain
P23935	NDUFA5	0.42	0.000	NADH dehydrogenase
P49951	CLTC	0.42	0.000	Clathrin heavy chain 1
Q3T0P6	PGK1	0.42	0.000	Phosphoglycerate kinase 1
Q95KZ6	ECH	0.42	0.000	Enoyl-CoA hydratase
Q5E983	EEF1B	0.42	0.000	Elongation factor 1-beta
Q3SZN8	RNH1	0.42	0.000	Ribonuclease/angiogenin inhibitor 1
Q9BGI1	PRDX5	0.41	0.000	Peroxiredoxin-5, mitochondrial
G5E531	TCP1	0.41	0.000	T-complex protein 1 subunit alpha
Q3YJL8	BoLA	0.40	0.000	MHC class I antigen
Q29RZ0	ACAT1	0.40	0.000	Acetyl-CoA acetyltransferase
Q17QY3	NIPSNAP3A	0.40	0.005	Nipsnap homolog 3A
Q5W5U3	HK1	0.40	0.015	Hexokinase 1
Q3T171	RPL36	0.39	0.000	60S ribosomal protein L36
P46193	ANXA1	0.39	0.000	Annexin A1
F1MTC2	QARS	0.39	0.000	Glutamine--tRNA ligase
Q3ZBS8	TIMM8A	0.39	0.000	Mitochondrial import inner membrane translocase
G3MZD8		0.39	0.000	Uncharacterized protein
P50397	RABGDIB	0.38	0.000	Rab GDP dissociation inhibitor beta
P80311	PPIB	0.38	0.000	Peptidyl-prolyl cis-trans isomerase B
P26779	PSAP	0.38	0.000	Prosaposin (Proactivator polypeptide)
P19120	HSPA8	0.38	0.000	Heat shock cognate 71 kDa protein
P52193	CALR CRT	0.37	0.000	Calreticulin (CRP55) (Calregulin)
Q95M18	HSP90B1	0.36	0.000	Endoplasmin
Q3SZ65	EIF4A2	0.36	0.000	Eukaryotic initiation factor 4A-II
F1N3P2	USP5	0.36	0.015	Ubiquitinyl hydrolase 1
F1MKS3	TXNDC5	0.35	0.000	Thioredoxin domain-containing protein 5
A6QNL5	PDIA6	0.35	0.000	chromobox 1
Q861U5		0.35	0.000	Similar to 40S ribosomal protein S18
E1BHJ0		0.35	0.000	Profilin
P23356	UCHL1	0.35	0.000	Ubiquitin carboxyl-terminal hydrolase isozyme L1
A1L5A7	PDLIM7	0.34	0.000	Enigma protein (PDZ and LIM domain protein 7)
Q3SZ62	PGAM1	0.34	0.000	Phosphoglycerate mutase 1
A7Z057	YWHAG	0.34	0.000	14-3-3 protein gamma (YWHAG protein)
Q3ZC84	CNDP2	0.34	0.000	Cytosolic non-specific dipeptidase
Q5E971	TMED10	0.33	0.000	Transmembrane emp24 domain-containing protein 10
Q3ZCI9	CCT8	0.33	0.000	T-complex protein 1 subunit theta (TCP-1-theta)
Q2TBQ5	RPL7A	0.33	0.000	60S ribosomal protein L7a
Q27954	COPA	0.33	0.000	Coatomer subunit alpha
Q3ZBY4	ALDOC	0.33	0.004	Fructose-bisphosphate aldolase
Q0QEV5	RPL18	0.33	0.000	Ribosomal protein L18
C1K3N7	TMSB4X	0.32	0.000	Thymosin beta 4 X-linked
A3KN04	RPN1	0.32	0.000	Dolichyl-diphosphooligosaccharide--protein
P62935	PPIA	0.32	0.000	Peptidyl-prolyl cis-trans isomerase A
G8JKX9		0.32	0.000	Uncharacterized protein
O02717		0.32	0.000	Non-muscle myosin heavy chain
E1BHK2	ADD1	0.31	0.000	Adducin 1
F1N6Y1	GANAB	0.31	0.000	Glucosidase II alpha subunit
Q56JX8	RPS13	0.31	0.009	40S ribosomal protein S13
Q0II59	PDXK	0.31	0.000	Pyridoxal kinase

Q3SZG7	RPL13	0.31	0.000	60S ribosomal protein L13
Q3T165	PHB	0.31	0.000	Prohibitin
A1XED1		0.30	0.000	RPL7
P61157	ACTR3	0.30	0.000	Actin-related protein 3 (Actin-2)
F1MKZ5		0.30	0.000	40S ribosomal protein S6
Q3T0U3	ES1	0.30	0.000	Es1 protein
P62261	YWHAE	0.30	0.000	14-3-3 protein epsilon (14-3-3E)
P42899	RPLP2	0.30	0.000	60S acidic ribosomal protein P2
Q56JV9	RPS3A	0.30	0.000	40S ribosomal protein S3a
Q32PD5	RPS19	0.30	0.000	40S ribosomal protein S19
O77834	PRDX6	0.30	0.000	Peroxiredoxin-6
Q3ZCF0	DCTN2	0.30	0.000	Dynactin subunit 2
Q32LM0	SH3GLB1	0.30	0.000	Endophilin-B1
G8JKV5	RPL14	0.29	0.000	60S ribosomal protein L14
Q3T0I5	FIS1	0.29	0.023	Mitochondrial fission 1 protein (Fis1 homolog)
Q6EWQ7	EIF5A	0.29	0.000	Eukaryotic translation initiation factor 5A-1
A7Z014	TKT	0.29	0.000	TKT protein (Transketolase)
Q5E997	CAPZA2	0.29	0.000	F-actin-capping protein subunit alpha-2
A5D7E8	PDIA3	0.29	0.000	Protein disulfide-isomerase
Q5E947	PRDX1	0.29	0.000	Peroxiredoxin-1
Q0VCX2	HSPA5	0.29	0.000	Endoplasmic reticulum chaperone BiP
Q3T0X5	PSMA1	0.28	0.000	Proteasome subunit alpha type-1
Q58DM9	RPL27A	0.28	0.000	Ribosomal protein L27a
Q58DW0	RPL4	0.28	0.000	60S ribosomal protein L4
Q9BGI3	PRDX2	0.27	0.000	Peroxiredoxin-2
Q3T0S7	RTRAF	0.27	0.000	Chromosome 14 open reading frame 166 ortholog
A5PK63	RPS17	0.27	0.000	40S ribosomal protein S17
Q32PB9	RPL38	0.27	0.000	60S ribosomal protein L38
F8UZU9	RPSA	0.27	0.000	40S ribosomal protein SA
Q5E9E6	RPL10A	0.27	0.000	60S ribosomal protein L10a
P61286	PABPC1	0.27	0.000	Polyadenylate-binding protein 1
Q58DW5	RPL5	0.27	0.000	60S ribosomal protein L5
A6H769	RPS7	0.26	0.000	40S ribosomal protein S7
P19803	ARHGDIA	0.26	0.000	Rho GDP-dissociation inhibitor 1
Q862L6		0.26	0.000	Similar to ribosomal protein L12
Q3T087	RPL11	0.26	0.000	60S ribosomal protein L11
P17248	WARS	0.26	0.000	Tryptophan--tRNA ligase, cytoplasmic
Q58DQ3	RPL6	0.25	0.000	60S ribosomal protein L6
Q56K14	RPLP1	0.25	0.000	60S acidic ribosomal protein P1
Q862I1	RPL24	0.25	0.000	60S ribosomal protein L24
P53620	COPG1	0.25	0.000	Coatomer subunit gamma-1
F1N301	RPL22	0.25	0.000	Uncharacterized protein
Q861S4	RPL21	0.24	0.000	Similar to ribosomal protein L21
F1N6U4	SEPT9	0.24	0.005	Septin 9
B1H0W8	Rpl3	0.24	0.005	Ribosomal protein L3
Q0P569	NUCB1	0.24	0.013	Nucleobindin-1
Q3T0K2	CCT3	0.24	0.000	T-complex protein 1 subunit gamma
P26884	FKBP3	0.24	0.007	Peptidyl-prolyl cis-trans isomerase FKBP3
F1MZN7	SNX2	0.24	0.001	Sorting nexin-2
Q3T057	RPL23	0.24	0.000	60S ribosomal protein L23

G3X7D5	LOC787803	0.23	0.000	Uncharacterized protein
Q9BEH4		0.23	0.000	Ribosomal protein L9-like protein
Q27971	CAPN2	0.23	0.000	Calpain-2 catalytic subunit
Q2KHX6	CRYM	0.23	0.000	Ketimine reductase mu-crystallin
Q27970	CAPN1	0.22	0.010	Calpain-1 catalytic subunit
E1B748	HYOU1	0.22	0.000	Hypoxia up-regulated protein 1 precursor
A5D987	UBE2N	0.22	0.000	Ubiquitin-conjugating enzyme E2N-like
E1BBY7	HSPA4	0.22	0.000	Heat shock protein family A (Hsp70) member 4
Q3T035	ARPC3	0.22	0.000	Actin-related protein 2/3 complex subunit 3
F1MC48	IQGAP1	0.22	0.000	IQ motif containing GTPase activating protein 1
G3MWT9	AKAP12	0.21	0.003	A-kinase anchoring protein 12
Q3ZBD7	GPI	0.21	0.000	Glucose-6-phosphate isomerase (GPI)
P79134	ANXA6	0.21	0.000	Annexin A6 (Annexin VI) (Annexin-6)
E1BMW2	AP2A1	0.21	0.009	AP-2 complex subunit alpha
Q76I81	RPS12	0.21	0.000	40S ribosomal protein S12
Q3T0B7	RPS27L	0.21	0.000	40S ribosomal protein S27-like
A7E3S8	ST13	0.21	0.000	Heat shock 70kD protein binding protein
E1BJB1	TUBB2A	0.21	0.000	Tubulin beta chain
G3N0K3		0.21	0.000	40S ribosomal protein S4
F1MQ37	MYH9	0.20	0.000	Myosin heavy chain 9
Q3T169	RPS3	0.20	0.000	40S ribosomal protein S3 (EC 4.2.99.18)
G3N0I4	LAP3	0.19	0.000	Cytosol aminopeptidase
G8JKY0	RPS8	0.19	0.000	40S ribosomal protein S8
P61585	RHOA	0.19	0.000	Transforming protein RhoA (Gb) (p21)
Q32LE5	ASRGL1	0.19	0.005	Isoaspartyl peptidase/L-asparaginase
G5E6M8	RPS10	0.19	0.000	40S ribosomal protein S10
G5E5C8	TALDO1	0.19	0.000	Transaldolase (EC 2.2.1.2)
Q3T0X6	RPS16	0.19	0.000	40S ribosomal protein S16
Q5E988	RPS5	0.18	0.014	40S ribosomal protein S5
P52556	BLVRB	0.17	0.010	Flavin reductase (NADPH)
Q2HJI1	SDHA	0.17	0.021	Succinate dehydrogenase
E1BFV0	KPNB1	0.17	0.005	Karyopherin subunit beta 1
Q3MHL7	CCT6A	0.16	0.000	T-complex protein 1 subunit zeta
F1MRG7	ACTR2	0.16	0.000	Actin-related protein 2
G5E631	DDX3X	0.16	0.002	DEAD-box helicase 3, X-linked
Q3SYT9	PCBP2	0.16	0.000	Poly(RC) binding protein 2
Q2HJ81	TUBB6	0.16	0.000	Tubulin beta-6 chain
E1BDX8	DYNC1H1	0.16	0.010	Cytoplasmic dynein 1 heavy chain 1
P81947		0.16	0.000	Tubulin alpha-1B chain
Q3T0F7	MTPN	0.15	0.002	Myotrophin
Q9TS10		0.15	0.037	Dolichyl-diphosphooligosaccharide--protein
F1MEN8	PDIA4	0.15	0.000	Protein disulfide-isomerase A4
G3N2G7	MAP4	0.15	0.017	Microtubule-associated protein
G1K192	ARL3	0.15	0.000	ADP-ribosylation factor-like protein 3
Q0VCK0	ATIC PURH	0.15	0.000	Bifunctional purine biosynthesis protein
Q2NKU4	CARHSP1	0.15	0.000	Calcium regulated heat stable protein 1
F1N6C0	CALM2	0.15	0.000	Calmodulin
Q5E9A3	PCBP1	0.14	0.000	Poly(rC)-binding protein 1 (Alpha-CP1)
Q2HJ49	MSN	0.14	0.000	Moesin
P63103	YWHAZ	0.14	0.000	14-3-3 protein zeta/delta

B0JYL8	CFL1	0.14	0.000	Cofilin 1 (Non-muscle)
Q862B7		0.14	0.001	Similar to ribosomal protein S14
P00570	AK1	0.13	0.000	Adenylate kinase isoenzyme 1 (AK 1)
G3MXC8	ARPC5	0.13	0.027	Actin-related protein 2/3 complex subunit 5
Q56JU9	RPS24	0.13	0.017	40S ribosomal protein S24
Q0VCY8	PEA15	0.13	0.000	Phosphoprotein enriched in astrocytes 15
P60712	ACTB	0.12	0.000	Actin, cytoplasmic 1 (Beta-actin)
Q3SZJ7	LAMP2	0.12	0.000	Lysosomal-associated membrane protein 2
G5E5T5		0.11	0.000	Uncharacterized protein
Q3T0E0	ATOX1	0.11	0.008	Copper transport protein ATOX1
Q3SYV4	CAP1	0.11	0.003	Adenylyl cyclase-associated protein 1 (CAP 1)
E1BFB9	FAM114A1	0.11	0.003	Protein FAM114A2
P51122	ANP32A	0.10	0.001	Acidic leucine-rich nuclear phosphoprotein 32
Q76LV2	HSP90AA1	0.10	0.000	Heat shock protein HSP 90-alpha
F1N7T1	CYB5R3	0.09	0.034	NADH-cytochrome b5 reductase
Q2KJD0	TUBB5	0.08	0.000	Tubulin beta-5 chain
Q3ZCK2	RALA	0.08	0.005	RAS like proto-oncogene A (V-ral simian leukemia
Q1RMP3	CUTA	0.07	0.000	CutA divalent cation tolerance homolog (E. coli)
P81623	ERP29	0.07	0.012	Endoplasmic reticulum resident protein 29
E1B9K1	UBC	0.06	0.001	Polyubiquitin-C
P48616	VIM	0.06	0.000	Vimentin
F1N7Q7	COL4A2	0.05	0.048	Collagen alpha-2(IV) chain
F1MTV9	NPM1	0.05	0.010	Nucleophosmin
K4JDR8	A2M	-0.05	0.019	Alpha-2-macroglobulin variant 5
Q3T0D0	HNRNPK	-0.07	0.003	Heterogeneous nuclear ribonucleoprotein K
Q2KIS8	SNRPD3	-0.08	0.000	Small nuclear ribonucleoprotein D3 polypeptide 1
G3X8G9		-0.08	0.007	Uncharacterized protein
A5D7D1	ACTN4	-0.08	0.000	Alpha-actinin-4 (Non-muscle alpha-actinin 4)
E1BNQ9	HNRNPUL2	-0.09	0.003	Uncharacterized protein
A6H744	UBE2I	-0.09	0.000	SUMO-conjugating enzyme UBC9
Q8HYY3		-0.09	0.004	L-caldesmon
F1MPD1	MRC2	-0.09	0.002	Mannose receptor C type 2
V6F7P3	PAFAH1B2	-0.09	0.000	Platelet-activating factor acetylhydrolase IB
F1N7N1	QKI	-0.09	0.000	QKI, KH domain containing RNA binding
F1MIH2	SEPT7	-0.09	0.026	Septin-7
Q3MHN0	PSMB6	-0.10	0.021	Proteasome subunit beta type-6
Q5E946	PARK7	-0.11	0.002	Protein/nucleic acid deglycase DJ-1
A5PJE3	FGA	-0.12	0.000	Fibrinogen alpha chain
F1MFT4	HNRNPU	-0.12	0.000	Heterogeneous nuclear ribonucleoprotein U
Q2NKY7	SEPT2	-0.12	0.000	Septin-2
Q2KIU3		-0.12	0.026	Protein HP-25 homolog 2
P62833	RAP1A	-0.12	0.000	Ras-related protein Rap-1A (GTP-binding protein)
G3N3L9	LOC613363	-0.12	0.000	Uncharacterized protein
E1BEG2	HNRNPA3	-0.12	0.000	Heterogeneous nuclear ribonucleoprotein A3
B0JYQ0	ALB	-0.13	0.050	ALB protein
Q3T0C7	STMN1	-0.13	0.006	Stathmin
Q3SX33	THY1	-0.13	0.010	Thy-1 cell surface antigen
G1K208	ACSF2	-0.14	0.003	Acyl-CoA synthetase family member 2
Q3ZC44	HNRNPAB	-0.14	0.000	Heterogeneous nuclear ribonucleoprotein A/B
A6H6Y0	HNRNPD	-0.14	0.000	Heterogeneous nuclear ribonucleoprotein

E1BMD6	LPP	-0.14	0.000	LIM domain containing preferred translocation protein
Q7SIH1	A2M	-0.14	0.000	Alpha-2-macroglobulin (Alpha-2-M)
P62871	GNB1	-0.14	0.011	Guanine nucleotide-binding protein G(I)
Q2KJC7	POSTN	-0.14	0.043	Periostin (Periostin variant 7)
E1BQ37	SFPQ	-0.14	0.001	Splicing factor proline and glutamine rich
F1MER7	HSPG2	-0.14	0.000	Heparan sulfate proteoglycan 2
P00515	PRKAR2A	-0.14	0.000	cAMP-dependent PK type II-alpha regulatory subunit
A6QR15	TPM4	-0.15	0.000	LOC535277 protein (Tropomyosin 4)
Q9GLX9	SPON1	-0.15	0.001	Spondin-1 (F-spondin)
Q3ZC07	ACTC1	-0.15	0.000	Actin, alpha cardiac muscle 1
Q2KJ32	SELENBP1	-0.15	0.002	Methanethiol oxidase (MTO)
F1MSI2	AGRN	-0.15	0.000	Agrin
Q3SZZ9	FGG	-0.15	0.000	FGG protein (Fibrinogen gamma-B chain)
F1MAV0	FGB	-0.16	0.000	Fibrinogen beta chain
Q5E9E2	MYL9	-0.16	0.000	Myosin regulatory light polypeptide 9
P02253	HIST1H1C	-0.17	0.004	Histone H1.2
F1MHR6	KHSRP	-0.17	0.000	KH-type splicing regulatory protein
Q28009	FUS	-0.17	0.000	RNA-binding protein FUS (Protein pigpen)
D3JUI8	RBMX	-0.17	0.001	RNA-binding motif protein, X chromosome
F1MF97	NID2	-0.18	0.000	Nidogen 2
P11017	GNB2	-0.18	0.002	Guanine nucleotide-binding protein G(I)
Q5KR48	TPM2	-0.18	0.000	Tropomyosin beta chain
F1N7I5	NUMA1	-0.19	0.003	Nuclear mitotic apparatus protein 1
Q3B7N2	ACTN1	-0.19	0.000	Alpha-actinin-1
P21809	BGN	-0.20	0.002	Biglycan (Bone/cartilage proteoglycan I)
A7MB21	FERMT2	-0.20	0.006	FERMT2 protein (Fermitin family member 2)
E1B8P9	MAPK3	-0.20	0.006	Mitogen-activated protein kinase
O02675	DPYSL2	-0.20	0.000	Dihydropyrimidinase-related protein 2
F1MWK8	PTK7	-0.20	0.013	Protein tyrosine kinase 7 (inactive)
Q08E18	HDGF	-0.21	0.001	HDGF protein
G5E505	ILK	-0.21	0.000	Integrin-linked protein kinase
A7MBI5	DPYSL3	-0.21	0.000	DPYSL3 protein (Dihydropyrimidinase like 3)
A6QP73	DDX31	-0.21	0.000	RNA helicase
V6F957	TAGLN	-0.21	0.000	Transgelin
Q3B7M5	LASP1	-0.21	0.000	LIM and SH3 domain protein 1 (LASP-1)
Q3SZR8	SRSF3	-0.21	0.000	Serine/arginine-rich splicing factor 3
P60902	S100A10	-0.21	0.000	Protein S100-A10 (Calpactin I light chain)
F1N2L9	ALDH9A1	-0.21	0.032	4-trimethylaminobutyraldehyde dehydrogenase
Q2KJH0	TMOD3	-0.21	0.020	Tropomodulin 3
A7YWB6	TGFBI	-0.22	0.000	Transforming growth factor-beta-induced protein
A3KMV6	HNRNPR	-0.22	0.045	Heterogeneous nuclear ribonucleoprotein
Q3MHY1	CSRP1	-0.22	0.000	Cysteine and glycine-rich protein 1
Q28824	MYLK	-0.23	0.000	Myosin light chain kinase, smooth muscle (MLCK)
Q3SYU6	CNN2	-0.23	0.000	Calponin-2
F1MYC9	SPTBN1	-0.23	0.000	Spectrin beta chain
P79136	CAPZB	-0.23	0.016	F-actin-capping protein subunit beta (CapZ beta)
F1MK08	TPP1	-0.23	0.001	Tripeptidyl-peptidase 1
Q5EA67	ITIH4	-0.24	0.000	Inter-alpha (Globulin) inhibitor H4
A0A0F7RPX(	MIF	-0.24	0.000	Macrophage migration inhibitory factor
Q9BGU1	BTHRG	-0.24	0.000	Histidine-rich glycoprotein

Q5E9A1	NACA	-0.24	0.007	Nascent polypeptide-associated complex subunit a
F1MD77	LAMC1	-0.24	0.001	Laminin subunit gamma 1
A7MBH9	GNAI2	-0.24	0.000	G protein subunit alpha i2
P06868	PLG	-0.25	0.000	Plasminogen
Q2KIV8	GSTM3	-0.25	0.000	Glutathione S-transferase
A0A0F6QNI	C3	-0.27	0.000	Complement component 3
Q2KIS7	CLEC3B	-0.27	0.000	Tetranectin (TN)
F1N1I6	GSN	-0.27	0.000	Gelsolin
Q3MHL4	AHCY	-0.27	0.000	Adenosylhomocysteinase (AdoHcyase)
Q5EAD2	PHGDH	-0.27	0.018	D-3-phosphoglycerate dehydrogenase
F1MMK9	AMBP	-0.27	0.000	Protein AMBP
F1MXI4	MATR3	-0.27	0.000	Uncharacterized protein
E1BFB0	SPTAN1	-0.28	0.000	Spectrin alpha
G5E5N3	DHX9	-0.28	0.000	ATP-dependent RNA helicase A
A6QLZ3	ARSB	-0.28	0.001	ARSB protein (Arylsulfatase B)
Q148F8	HSPB6	-0.28	0.006	Heat shock protein beta-6 (HspB6)
Q3SZV7	HPX	-0.28	0.000	Hemopexin
F1N169	FLNA	-0.28	0.000	Filamin A
E1BNE7	CAVIN1	-0.29	0.000	Caveolae associated protein 1
F1N789	VCL	-0.29	0.000	Vinculin
Q27966	MYO1C	-0.29	0.000	Unconventional myosin-Ic
A6QPP2	SERPIND1	-0.30	0.000	SERPIND1 protein (Serpin family D member 1)
P02769	ALB	-0.31	0.000	Serum albumin (BSA) (allergen Bos d 6)
Q9XSA7	CLIC4	-0.31	0.000	Chloride intracellular channel protein 4
E1BA17	COL14A1	-0.31	0.000	Collagen type XIV alpha 1 chain
P13696	PEBP1	-0.31	0.000	Phosphatidylethanolamine-binding protein 1
P68432		-0.32	0.000	Histone H3.1
E1BH06		-0.32	0.000	Uncharacterized protein
P60661	MYL6	-0.32	0.000	Myosin light polypeptide 6
E1BB17	HNRNPH3	-0.32	0.000	Heterogeneous nuclear ribonucleoprotein H3
E1BHR3	HDGFL3	-0.32	0.001	Hepatoma-derived growth factor-related protein 3
Q5KR49	TPM1	-0.33	0.000	Tropomyosin alpha-1 chain
Q2KIT0		-0.33	0.000	Protein HP-20 homolog
Q2KIF2	LRG1	-0.33	0.000	Leucine-rich alpha-2-glycoprotein 1
E1BI02	FMOD	-0.33	0.000	Fibromodulin
Q8WMQ6		-0.33	0.000	Filamin A (Fragment)
A6QM11	TRH	-0.33	0.000	Pro-thyrotropin-releasing hormone
Q3MHN5	GC	-0.33	0.000	Vitamin D-binding protein
Q3MHZ4	TGFB1I1	-0.33	0.000	Transforming growth factor beta-1-induced protein
F1N401	COL12A1	-0.33	0.000	Collagen alpha-1(XII) chain
I7CT57		-0.34	0.000	Vitamin D binding protein
Q08DB4	CPNE1	-0.34	0.000	Copine-1 (Copine I)
Q08DJ3	FUBP1	-0.34	0.013	Far upstream element (FUSE) binding protein 1
P28801	GSTP1	-0.34	0.000	Glutathione S-transferase P
F6Q9S4	PRKAR2B	-0.35	0.000	cAMP-dependent PK type II-beta regulatory subunit
F1MNV5	KNG1	-0.35	0.000	Kininogen-1
Q95121	SERPINF1	-0.35	0.000	Pigment epithelium-derived factor (PEDF) (Serpin)
P01044	KNG1	-0.35	0.000	Kininogen-1
G3MZ95	FHL1	-0.35	0.000	Four and a half LIM domains 1
Q29443	TF	-0.36	0.000	Serotransferrin (Transferrin)

P81644	APOA2	-0.36	0.000	Apolipoprotein A-II
F6QVC9	ANXA5	-0.36	0.000	Annexin
Q32S29	H2B	-0.36	0.000	Histone H2B
Q28085	CFH HF1	-0.36	0.000	Complement factor H (H factor 1)
O62654	DES	-0.36	0.000	Desmin
Q5E962	AKR1B1	-0.37	0.000	Aldo-keto reductase family 1, member B1
Q27991	MYH10	-0.37	0.000	Myosin-10 (Cellular myosin heavy chain, type B)
Q3T106	SRSF7	-0.37	0.000	Serine/arginine-rich splicing factor 7
G3MYZ3	AFM	-0.38	0.000	Afamin (Alpha-albumin)
F1MWN3	NID1	-0.38	0.000	Nidogen 1
E1BF47	TPR	-0.38	0.000	Translocated promoter region, nuclear basket protein
F1MCK2	AHNAK	-0.38	0.000	AHNAK nucleoprotein
Q27965	HSPA1B	-0.39	0.000	Heat shock 70 kDa protein 1B
F1MNT4	LAMB1	-0.39	0.000	Laminin subunit beta 1
Q5GN72	AGP	-0.39	0.000	Alpha-1-acid glycoprotein
P02453	COL1A1	-0.39	0.000	Collagen alpha-1(I)
A8E644	DCLK1	-0.39	0.000	DCLK1 protein (Doublecortin like kinase 1)
F1MSQ9	TMPO	-0.40	0.000	Thymopoietin
P62739	ACTA2	-0.40	0.000	Actin, aortic smooth muscle (Alpha-actin-2)
I6X9I8		-0.40	0.027	Annexin
E1B8N6	LMNB1	-0.40	0.000	Lamin B1
G3N0V0		-0.40	0.000	Uncharacterized protein
Q32PI4	CFI	-0.41	0.000	Complement factor I
Q2KJF1	A1BG	-0.41	0.000	Alpha-1B-glycoprotein
P81287	ANXA5	-0.41	0.000	Annexin A5
O46375	TTR	-0.41	0.000	Transthyretin
P34955	SERPINA1	-0.41	0.000	Alpha-1-antiproteinase (Alpha-1-antitrypsin)
Q2TBR3	SET	-0.42	0.000	SET nuclear oncogene
P17690	APOH	-0.42	0.000	Beta-2-glycoprotein 1 (Apolipoprotein H) (Apo-H)
P00735	F2	-0.42	0.000	Prothrombin
E1BMJ0	SERPING1	-0.42	0.000	Factor XIIa inhibitor precursor
Q29437		-0.42	0.000	Primary amine oxidase, liver isozyme
F1N076	CP	-0.43	0.000	Ceruloplasmin
A5D7U9	DCPS	-0.43	0.001	DCPS protein (m7GpppX diphosphatase)
Q3MHN2	C9	-0.43	0.000	Complement component C9
Q2HJ65	H2AFY	-0.43	0.000	Core histone macro-H2A
Q148J6	ARPC4	-0.44	0.018	Actin-related protein 2/3 complex subunit 4
G5E6N7	CBX1	-0.44	0.000	Chromobox 1
F1MZ96		-0.44	0.000	Uncharacterized protein
Q32LA7	H2AFV	-0.45	0.000	Histone H2A.V (H2A.F/Z)
Q1LZF9	MYL9	-0.45	0.000	Myosin regulatory light polypeptide 9
P10103	HMGB1	-0.45	0.000	High mobility group protein B1
G3X807		-0.45	0.000	Histone H4
F1N3A1	THBS1	-0.46	0.000	Thrombospondin-1
E9RHW1	HSPB1	-0.46	0.000	Heat shock 27kDa protein 1
B0JYN6	AHSG	-0.47	0.000	Alpha-2-HS-glycoprotein
E1BGN3	HIST2H3D	-0.47	0.004	Histone H3
Q05443	LUM LDC	-0.47	0.000	Lumican
F1MKG2	COL6A2	-0.48	0.000	Collagen type VI alpha 2 chain
Q9N0V4	GSTM1	-0.48	0.000	Glutathione S-transferase Mu 1

Q2KIX7		-0.49	0.000	Protein HP-25 homolog 1
F1MSZ6	SERPINC1	-0.50	0.000	Antithrombin-III
Q3ZBS7	VTN	-0.50	0.000	Vitronectin
Q2TA04	GPNMB	-0.50	0.000	Glycoprotein (Transmembrane) nmb
P21793	DCN	-0.51	0.000	Decorin (Bone proteoglycan II) (PG-S2)
P61283	BANF1	-0.51	0.000	Barrier-to-autointegration factor
Q58D62	FETUB	-0.51	0.000	Fetuin-B
A0A0A0MPA0		-0.52	0.000	Uncharacterized protein
E1BI98	COL6A1	-0.52	0.000	Collagen type VI alpha 1 chain
P81187	CFB BF	-0.52	0.000	Complement factor B
Q687I9	EDIP	-0.53	0.000	Purine nucleoside phosphorylase
E1BB91	COL6A3	-0.53	0.000	Collagen type VI alpha 3 chain
Q3T0W4	PPP1R7	-0.53	0.000	Protein phosphatase 1 regulatory subunit 7
F1MY85	C5	-0.53	0.000	Complement C5a anaphylatoxin
F1MYG5	LMNA	-0.54	0.000	Lamin A/C
F1MUK3	IGFBP6	-0.54	0.000	Insulin-like growth factor-binding protein 6
P02465	COL1A2	-0.55	0.000	Collagen alpha-2(I)
E1BLS8	EMILIN1	-0.56	0.000	Elastin microfibril interfacer 1
F1N453	HIST2H2BE	-0.56	0.000	Histone H2B
A6QLN9	VWA1	-0.57	0.000	von Willebrand factor A domain-containing protein
Q08DU9	HP1BP3	-0.57	0.000	Heterochromatin protein 1-binding protein 3
G5E6E8		-0.57	0.000	Histone H2B
F2Z4I6	HIST2H2A	-0.57	0.000	Histone H2A
Q2KJ47	EHD2	-0.58	0.000	EH-domain containing 2
Q3SYZ8	PDLIM3	-0.59	0.000	PDZ and LIM domain protein 3
A6QLN6	MYH11	-0.60	0.000	MYH11 protein
P19427	DPT	-0.60	0.000	Dermatopontin
G3N131	HIST1H1A	-0.60	0.000	Histone H1.1
F1MMU4	H1FX	-0.61	0.000	H1 histone family member X
P17697	CLU	-0.61	0.000	Clusterin
Q862M5		-0.62	0.000	Similar to acidic ribosomal phosphoprotein PO
Q17QG8	H2AFX	-0.64	0.000	Histone H2A
G1K238	COL4A1	-0.65	0.000	Collagen alpha-1(IV) chain
Q1RMN8	IGL	-0.66	0.000	Immunoglobulin light chain lambda
F1MJA7	CYP17A1	-0.66	0.000	Steroid 17-alpha-hydroxylase/17,20 lyase
G3N0V2	KRT1	-0.66	0.000	Keratin 1
P55918	MFAP4	-0.69	0.000	Microfibril-associated glycoprotein 4
Q3T085	OGN	-0.70	0.000	OGN protein
Q29S21	KRT7	-0.71	0.000	Keratin, type II cytoskeletal 7 (Cytokeratin-7)
F1MVK1		-0.71	0.000	Uncharacterized protein
F1MEG3	LAMA1	-0.72	0.001	Laminin subunit alpha 1
F2Z4J1	HIST1H2AC	-0.72	0.000	Histone H2A
Q29RQ1	C7	-0.74	0.000	Complement component C7
A6QNJ7	PGM5	-0.75	0.000	PGM5 protein (Phosphoglucomutase 5)
V6F9A2	APOA1	-0.75	0.000	Apolipoprotein A-I preproprotein
M0QVY0	KRT6A	-0.76	0.000	Uncharacterized protein
Q0VCP3	OLFML3	-0.77	0.000	Olfactomedin-like protein 3
F1N583	OLFML1	-0.77	0.000	Olfactomedin like 1
F1N4K8	FBN1	-0.78	0.000	Fibrillin-1 (MP340)
A7E307	DDX17	-0.80	0.001	DDX17 protein (DEAD-box helicase 17)

P50291	FST	-0.82	0.000	Fibrinogen alpha chain
Q0IIJ2	H1F0	-0.82	0.000	Histone H1.0
E1BDK6	LAMB2	-0.82	0.000	Laminin subunit beta 2
A6QQQ3	PRELP	-0.82	0.000	Proline/arginine-rich end leucine-rich repeat protein
F1N6I7	VCAN	-0.90	0.000	Versican core protein
Q8HYJ2	BLT	-0.93	0.000	Tryptase
Q17QE2	LMCD1	-0.96	0.000	LIM and cysteine-rich domains protein 1
Q1LZ92	H2AFY2	-0.98	0.000	Core histone macro-H2A
F6Q751	GSTM2	-0.99	0.000	Uncharacterized protein
Q3ZEJ6	SERPINA3-3	-1.00	0.000	Serpin A3-3
Q8HZY1	SERPINE2	-1.01	0.000	Serine protease inhibitor clade E member 2
V6F9A3	ApoC3	-1.02	0.000	Apolipoprotein C-III
O18977	TN-X	-1.12	0.000	Tenascin-X
F1MNL7	PTI	-1.15	0.000	Uncharacterized protein
Q9TTE1	SERPINA3A	-1.23	0.000	Serpin A3-1
Q3MHP6	EIF4B	-1.24	0.000	Eukaryotic translation initiation factor 4B
Q5E9F8	H3F3A/B	-1.46	0.000	Histone H3.3
Q3ZBN5	ASPN	-2.00	0.000	Asporin
**log2FC*=** log2 Fold Change in post-pubertal hiefers as compared to pre-pubertal heifers				
**Protein ID*=** Uniprot_accession protein ID				
