# Supplementary material for: Proteomics Recapitulates Ovarian Proteins Relevant to Puberty and Fertility in Brahman Heifers (Bos indicus L.)
Source: Genes (Basel). 2019 Nov 12;10(11):923. doi: 10.3390/genes10110923 (PMC6895798; doi:10.3390/genes10110923)
Supplement: Supplementary file 1 [file genes-10-00923-s001.zip › Table S2.pdf]

Supp. Table S2: Pathways and differential abundant proteins in post-pubertal heifers, as compared to pre-pubertal, involved in ovarian functions at puberty

No	Pathways	DA Proteins (Uniprot-Accession)
**Up-regulated Proteins Based Enriched Pathways**		
1	Ribosome	Q3T0B7, Q5E9E6, Q3T087, Q3SZG7, G8JKV5, Q0QEV5, Q861S4, F1N301, Q3T057, Q862I1, P61356, Q58DM9, Q3T0L7, Q3T171, Q32PB9, Q58DW0, Q58DW5, Q58DQ3, Q2TBQ5, G5E6M8, Q76I81, Q56JX8, Q3T0X6, A5PK63, Q32PD5, Q56JU9, Q3T169, Q56JV9, Q5E988, F1MKZ5, A6H769, G8JKY0, F8UZU9, Q56K14, P42899
2	Metabolic Pathways	A6QR14, Q3ZC79, Q0VCK0, Q32PF2, Q7JAT2, P13621, F1MLB8, P00829, P05630, P05631, P13620, Q28851, P02721, Q3ZC84, P42028, Q3SZE6, P23935, Q02827, Q01321, Q29RZ0, Q1JPB6, Q3ZC41, P20004, F1MH57, Q3ZBF6, P00570, P08166, P48644, A6QLL8, Q3ZBY4, Q29RK1, A3KMX9, Q8M444, P00423, P00429, P00426, P00125, P11179, F1MB08, F1N647, Q148D3, F1MMK2, Q3ZBD7, F1N6Y1, P33097, P12344, F1MTC2, P10096, Q5W5U3, P14893, Q2KJC5, Q3SZ00, A5D9E7, O02691, Q0QEQ4, Q9XSG3, P41563, F1MTJ9, G3N0I4, Q3T145, Q32LG3, P11024, A1A4N9, Q3T0Q4, O77834, Q3ZCI4, Q3T0P6, Q3SZ62, Q6VBM2, Q0VCM4, Q0II59, F1MHB8, A3KN04, Q2HJI1, Q3MHX5, Q58DR8, G5E5C8, A7Z014, Q3MI02, P23004, P00126, P13272
3	Oxidative Phosphorylation	Q7JAT2, P13621, F1MLB8, P00829, P05630, P05631, P13620, Q28851, P02721, P42028, Q3SZE6, P23935, Q02827, Q01321, Q8M444, P00423, P00429, P00426, P00125, Q2HJI1, Q3MI02, P23004, P00126, P13272

**Down-regulated Proteins Based Enriched Pathways**		
1	Complement and Coagulation Cascade	K4JDR8, P00735, A0A0F6QNP7, E1BH06, F1MY85, Q29RQ1, Q3MHN2, P81187, Q28085, Q32PI4, A5PJE3, F1MAV0, Q3SZZ9, P01044, P06868, P34955, F1MSZ6, A6QPP2, E1BMJ0
2	Systemic Lupus Erythematosus	Q32LA7, Q17QG8, Q2HJ65, Q1LZ92, Q5E9F8, Q3B7N2, A5D7D1, A0A0F6QNP7, E1BH06, F1MY85, Q29RQ1, Q3MHN2, G3N3L9, Q32S29, F2Z4J1, F2Z4I6, F1N453, E1BGN3, Q2KIS8
3	Focal Adhesion	P62833, Q3B7N2, A5D7D1, P02453, P02465, G1K238, E1BI98, F1MKG2, E1BB91, F1N169, G5E505, F1MEG3, F1MNT4, F1MD77, E1BDK6, E1B8P9, Q5E9E2, Q28824, O18977, F1N3A1, F1N789, Q3ZBS7
4	Extracellular Matrix Receptor Interaction	F1MSI2, P02453, P02465, G1K238, E1BI98, F1MKG2, E1BB91, F1MER7, F1MEG3, F1MNT4, F1MD77, E1BDK6, O18977, F1N3A1, Q3ZBS7
5	Alcoholism	A7MBH9, P62871, P11017, Q32LA7, Q17QG8, Q2HJ65, Q1LZ92, Q5E9F8, G3N3L9, Q32S29, F2Z4J1, F2Z4I6, F1N453, E1BGN3, E1B8P9
**Whole DA Proteins Based Functional Clusters**		
1	Glycolysis	A6QLL8, Q3ZBY4, F1MB08, Q3ZBD7, P10096, Q5W5U3, Q3T0P6, Q3SZ62, Q3ZC87
2	TCA Cycle	Q32PF2, P20004, Q29RK1, P11179, Q148D3, Q9XSG3, P41563, Q3T145, Q32LG3, Q2HJI1, Q3MHX5, Q58DR8
3	Pentose Phosphate Pathway	A6QLL8, Q3ZBY4, F1MMK2, Q3ZBD7, Q3ZCI4, G5E5C8, A7Z014

4	Oxidative Phosphorylation	Q7JAT2, P13621, F1MLB8, P00829, P05630, P05631, P13620, Q28851, P02721, P42028, Q3SZE6, P23935, Q02827, Q01321, Q8M444, P00423, P00429, P00426, P00125, Q2HJI1, Q3MI02, P23004, P00126, P13272
5	Fatty Acid Metabolism	Q29RZ0, Q1JPB6, Q3ZC41, Q3ZBF6, F1N647, Q2KJC5, Q3SZ00, A5D9E7, Q3ZCD7
6	Branched Chain Amino Acid Degradation	A5D9E7, F1MH57, F1N2L9, O02691, Q1JPB6, Q29RZ0, Q2KJC5, Q3SZ00, Q3ZBF6, Q3ZC41, Q3ZC79
7	Terpeniod Backbone Biosynthesis	Q3ZC79, Q29RZ0, Q1JPB6 , F1MTJ9
8	Cholesterol Metabolism	A6QR14, V6F9A2, P81644, P00432, A3KMX9, F1N7T1, P00257, Q148K8, F1MMK2, F1MTJ9
9	Cholesterol Efflux	P81644, V6F9A2, V6F9A3
10	Ovarian Steroidogenesis	A6QR14, Q3MHN5, Q3ZC41, V6F9A2, P81644, P00432, A3KMX9, F1MJA7, F1N7T1, P00257, Q148K8, F1MMK2, P14893, F1MTJ9, Q3ZCD7
11	Ooctye Maturation and Oocyte Meiosis	A7MBH9, Q76LV2, Q76LV1, E1B8P9, F1N6C0, A5D973, P62261, A7Z057, P63103, P00515, F6Q9S4, P08166
12	cAMP Signalling	A7MBH9, P62833, F1N6C0, E1B8P9, Q1LZF9, P61585
13	Estrogen Signalling	A7MBH9, F1N6C0, Q27965, Q76LV2, Q76LV1, Q95M18, P19120, E1B8P9
14	Cell Division	A7MBH9, P49951, G3N2G7, Q3T169, Q2NKY7, F1MIH2

15	Microtubule Processing	Q3ZCF0, E1BJB1, Q2HJ81, Q2KJD0
16	Regulation of Actin Cytoskeleton	F1MC48, P60712, Q3T035, Q148J6, G3MXC8, Q3B7N2, A5D7D1, P00735, B0JYL8, P31976, G5E5A9, F1N1I6, E1B8P9, Q2HJ49, Q1LZF9, Q28824, Q5E9E2, P61585, C1K3N7, F1N789
17	Focal Adhesion	P62833, P60712, Q3B7N2, A5D7D1, Q27971, P02453, P02465, G1K238, F1N7Q7, E1BI98, F1MKG2, E1BB91, G5E5A9, F1N169, G5E505, F1MEG3, F1MNT4, F1MD77, E1BDK6, E1B8P9, Q1LZF9, Q28824, Q5E9E2, P61585, O18977, F1N3A1, F1N789, Q3ZBS7
18	Cell Matrix Adhesion	F1MAV0, Q3SZZ9, F1MWN3, F1MF97, O18977
19	Protein Processing	A7Z066, Q27970, Q27971, P52193, F1ME65, P81623, F1N6Y1, Q27965, Q76LV2, Q76LV1, Q95M18, Q0VCX2, P19120, E1B748, A6H7J6, A5D7E8, F1MEN8, A6QNL5, A3KN04, F1MDC1, G3X757
20	Ribosome	Q5E9E6, Q3T087, Q3SZG7, G8JKV5, Q0QEV5, Q861S4, F1N301, Q3T057, Q862I1, P61356, Q58DM9, Q3T0L7, Q3T171, Q32PB9, Q58DW0, Q58DW5, Q58DQ3, Q2TBQ5,0 G5E6M8, Q76I81, Q56JX8, Q3T0X6, A5PK63, Q32PD5, Q56JU9, Q3T0B7 Q3T169, Q56JV9, Q5E988, F1MKZ5, A6H769, G8JKY0, F8UZU9, Q56K14, P42899
21	Translation	Q3SYU2, Q5E9E6, Q3T087, Q3SZG7, G8JKV5, Q0QEV5, Q861S4, Q3T057, Q862I1, P61356, Q58DM9, Q3T0L7, Q3T171, Q32PB9, Q58DW0, Q58DW5, Q58DQ3, Q2TBQ5, Q76I81, Q56JX8, Q3T0X6, A5PK63, Q32PD5, Q56JU9, Q3T0B7, Q3T169, Q56JV9, Q5E988,

		F1MKZ5, A6H769, G8JKY0, F8UZU9, F1MDC1, P79110, P12234, G3N3W3, P32007, A6QLN9
22	Nucleosome Assembly	Q0IIJ2, F1MMU4, Q2HJ65, Q1LZ92, Q5E9F8, Q2TBR3, P51122, Q08DU9, P10103, G3N3L9, Q32S29, G3N131, P02253, P68432, E1BGN3
23	Regulation of Transcription	P51122, Q2NKU4, Q08DB4, Q08E18, Q08DU9, F1N0I2, Q3T169, E1BQ37, G8JKZ8, F1MSQ9
24	Cell Redox Homeostasis	E1BJA2, P11024, Q5E947, Q9BGI3, P35705, Q9BGI1, O77834, A6H7J6, A5D7E8, F1MEN8, A6QNL5, Q5E936, G8JKZ8
25	Glutathione Metabolism	F1MMK2, Q9N0V4, F6Q751, Q28035, Q2KIV8, P28801, P00435, Q9XSG3, G3N0I4, Q3ZCI4, Q5E936
26	ECM Receptor Interaction	F1MSI2, P02453, P02465, G1K238, F1N7Q7, E1BI98, F1MKG2, E1BB91, G5E5A9, F1MER7, F1MEG3, F1MNT4, F1MD77, E1BDK6, O18977, F1N3A1, Q3ZBS7
